# Supplementary material for: Defining the root endosphere and rhizosphere microbiomes from the World Olive Germplasm Collection
Source: Sci Rep. 2019 Dec 31;9:20423. doi: 10.1038/s41598-019-56977-9 (PMC6938483; doi:10.1038/s41598-019-56977-9)
Supplement: Supplementary file 1 — Supplementary material. [file 41598_2019_56977_MOESM1_ESM.pdf]

# Defining the root endosphere and rhizosphere microbiomes from the World Olive Germplasm Collection

Antonio J. Fernández-González[a], Pablo J. Villadas[a], Carmen Gómez-Lama Cabanás[b], Antonio Valverde-Corredor[b], Angjelina Belaj[c], Jesús Mercado-Blanco[b], and Manuel Fernández-López[a*]

[a]Departamento de Microbiología del Suelo y Sistemas Simbióticos, Estación Experimental del Zaidín, Consejo Superior de Investigaciones Científicas (CSIC). Calle Profesor Albareda 1, 18008 Granada, Spain.

[b]Departamento de Protección de Cultivos, Instituto de Agricultura Sostenible, CSIC. Campus 'Alameda del Obispo' s/n, Avd. Menéndez Pidal s/n, 14004 Córdoba, Spain

[c]Área Mejora y Biotecnología, IFAPA-Centro Alameda del Obispo, Avda. Menéndez Pidal s/n, 14080 Córdoba, Spain

Figure S1. Normalized alpha diversity indices by compartment in the prokaryotic (a) and the fungal (b) communities. Endosphere (Endo), Rhizosphere (Rhizo) and Richness (Observed).

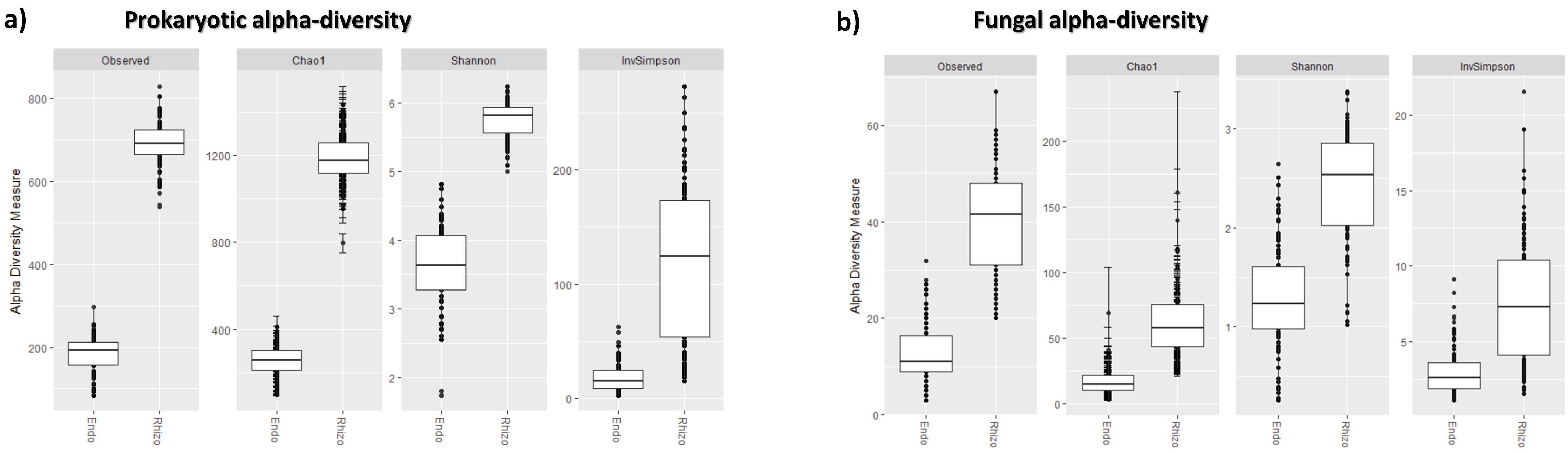

Figure S2. Microbial (bacterial a and b; fungal c and d) normalized alpha diversity indices of each sample clustered by cultivars in both compartments (a and c endosphere; b and d rhizosphere).

**a) Bacterial Endosphere**

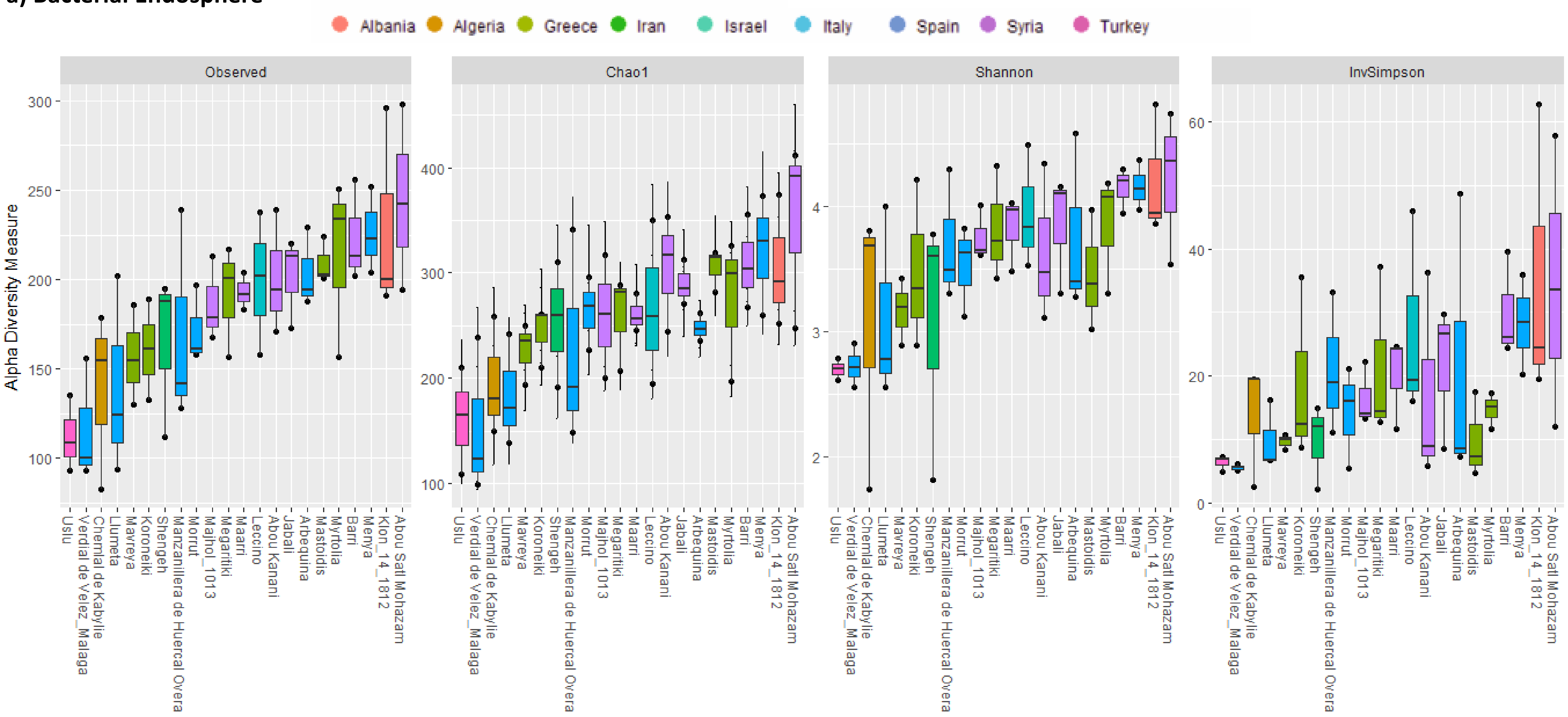

Figure S2. Microbial (bacterial a and b; fungal c and d) normalized alpha diversity indices of each sample clustered by cultivars in both compartments (a and c endosphere; b and d rhizosphere).

## b) Bacterial Rhizosphere

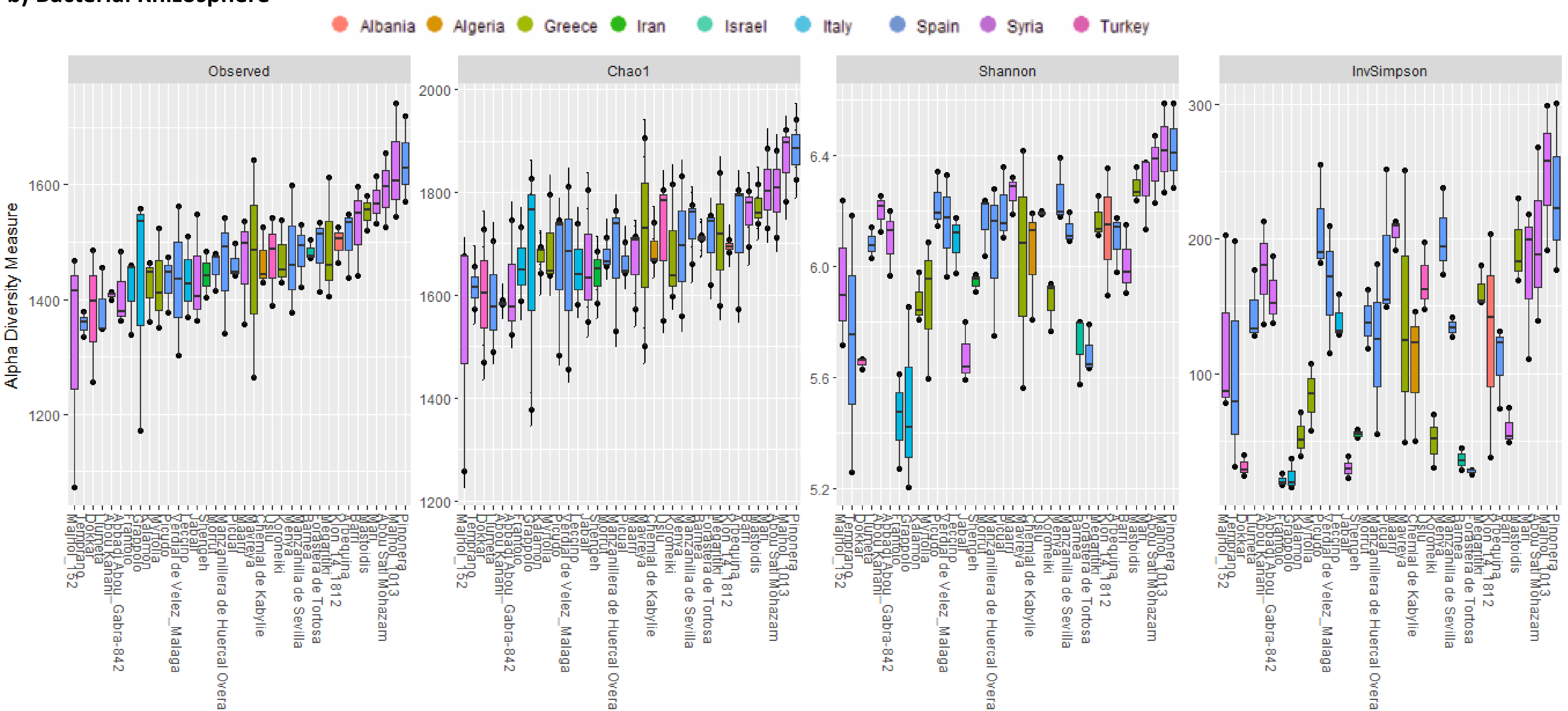

Figure S2. Microbial (bacterial a and b; fungal c and d) normalized alpha diversity indices of each sample clustered by cultivars in both compartments (a and c endosphere; b and d rhizosphere).

## c) Fungal Endosphere

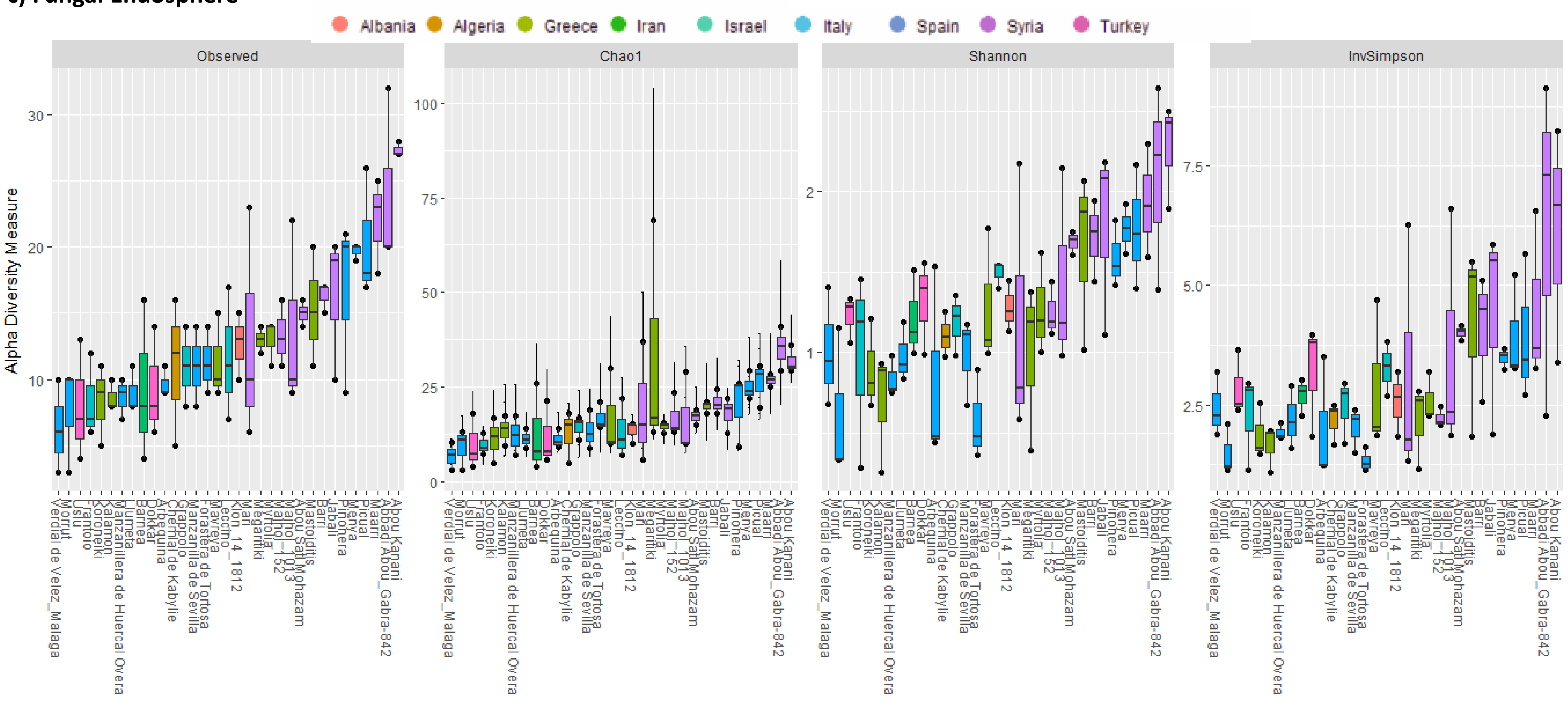

Figure S2. Microbial (bacterial a and b; fungal c and d) normalized alpha diversity indices of each sample clustered by cultivars in both compartments (a and c endosphere; b and d rhizosphere).

**d) Fungal Rhizosphere**

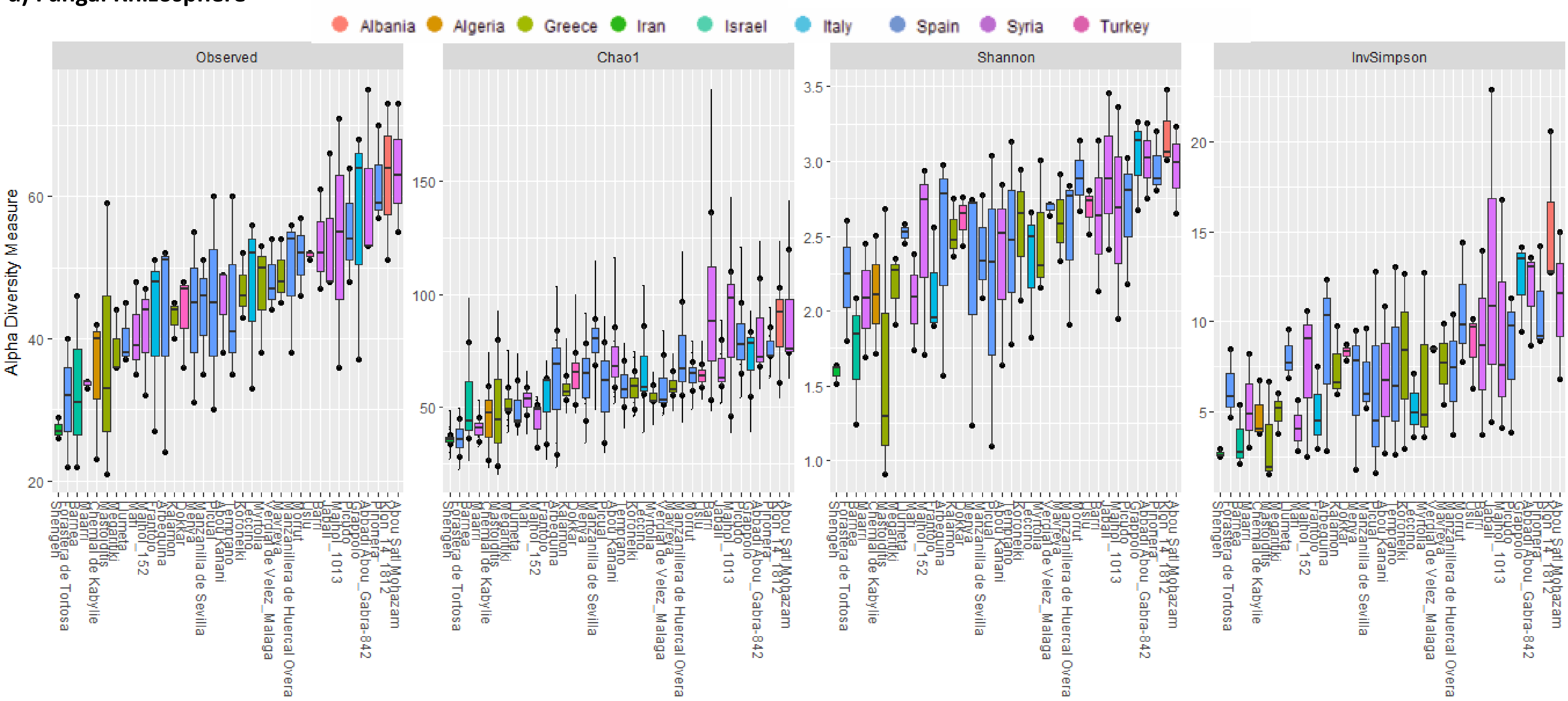

Figure S3. Statistically significant endophytic bacterial genera by cultivar (a, b) and the main bacterial genera in the endosphere (c).

a)

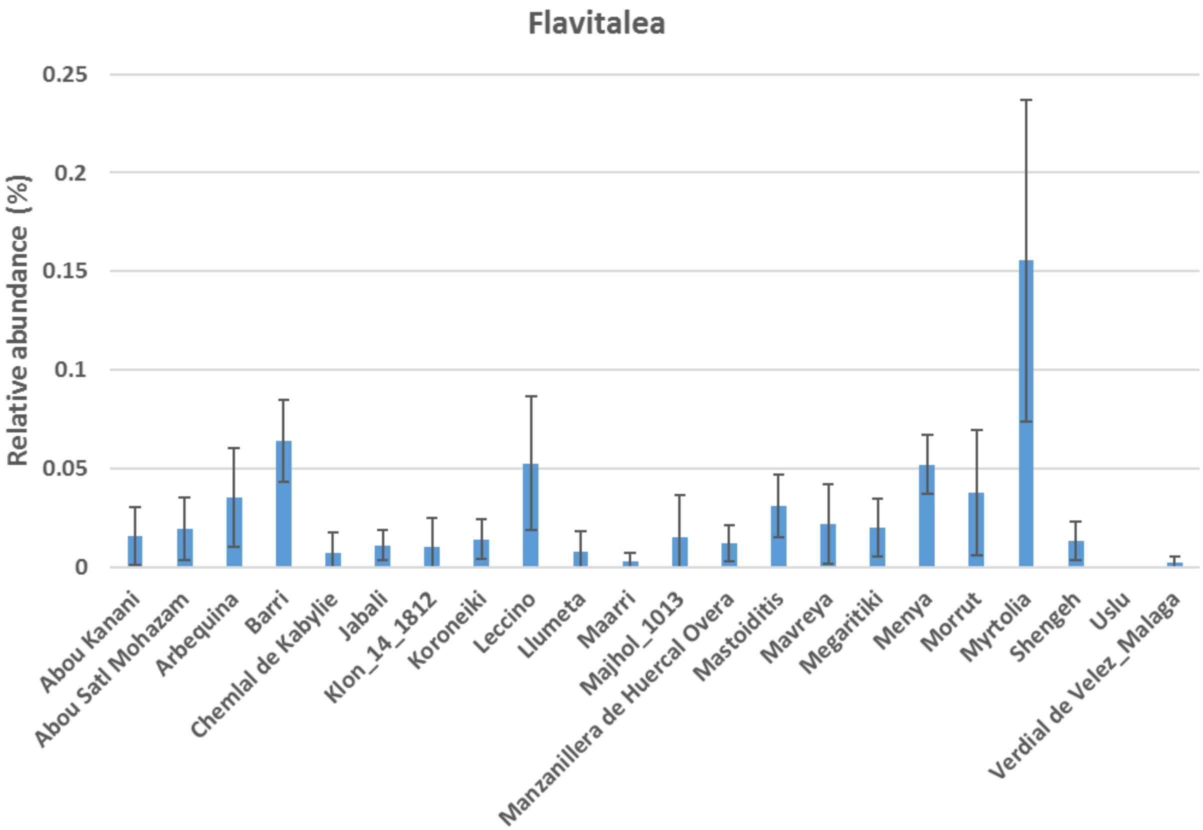


b)

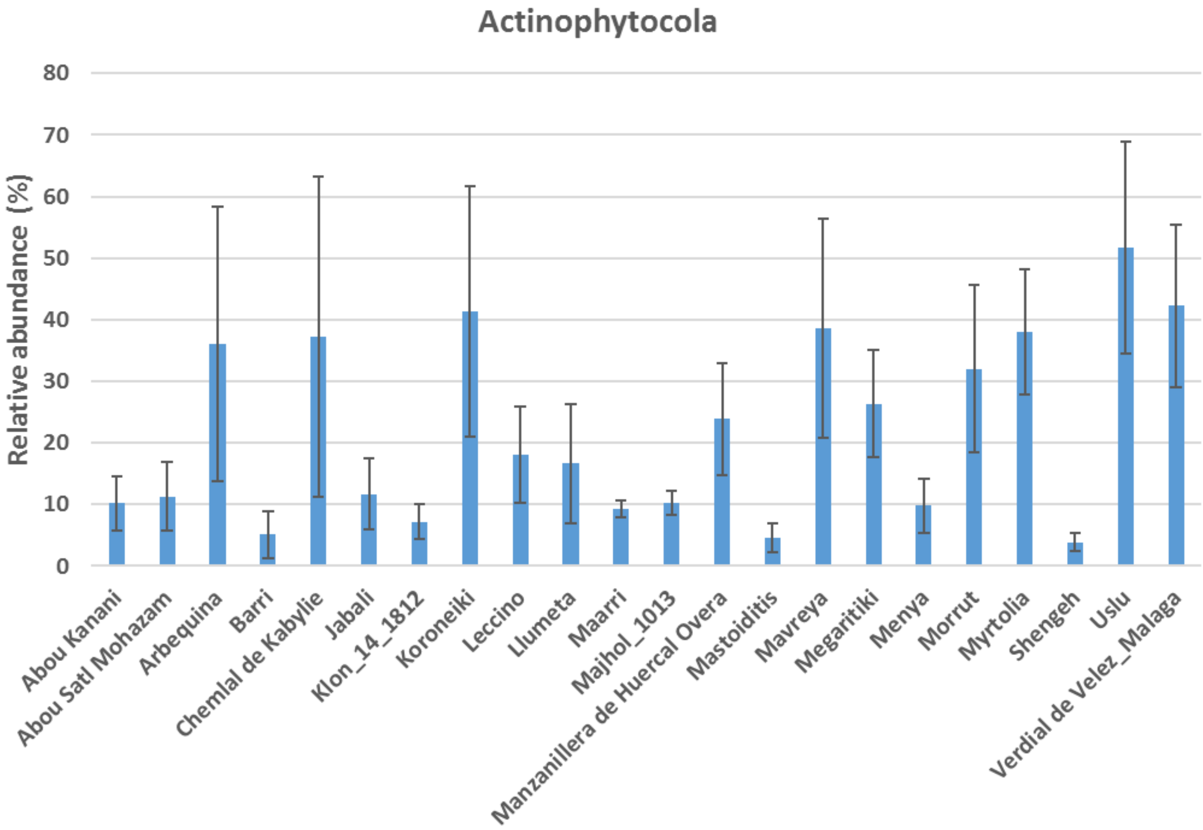

Figure S3. Statistically significant endophytic bacterial genera by cultivar (a, b) and the main bacterial genera in the endosphere (c).

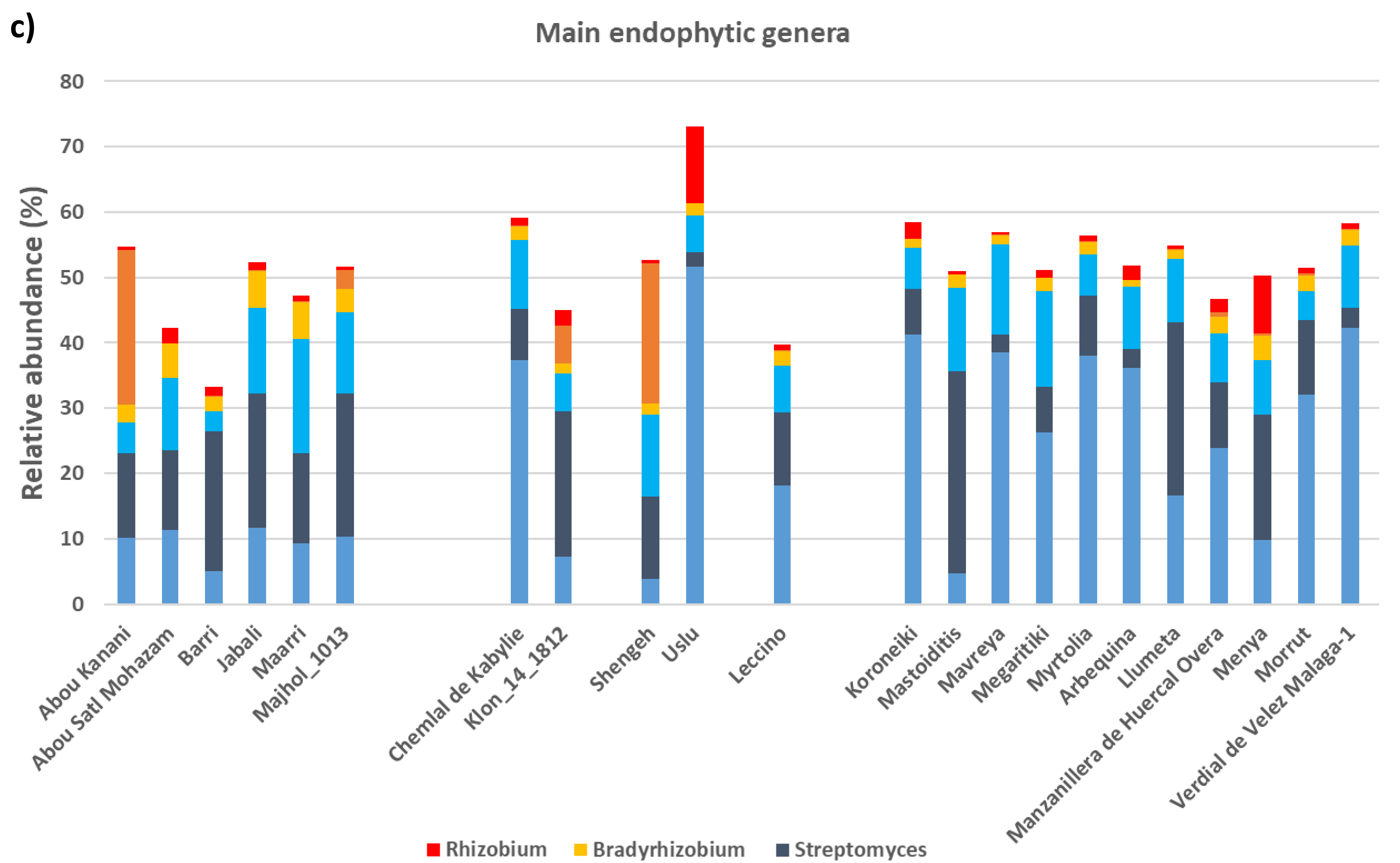

Figure S4. Main bacterial genera in the rhizosphere.

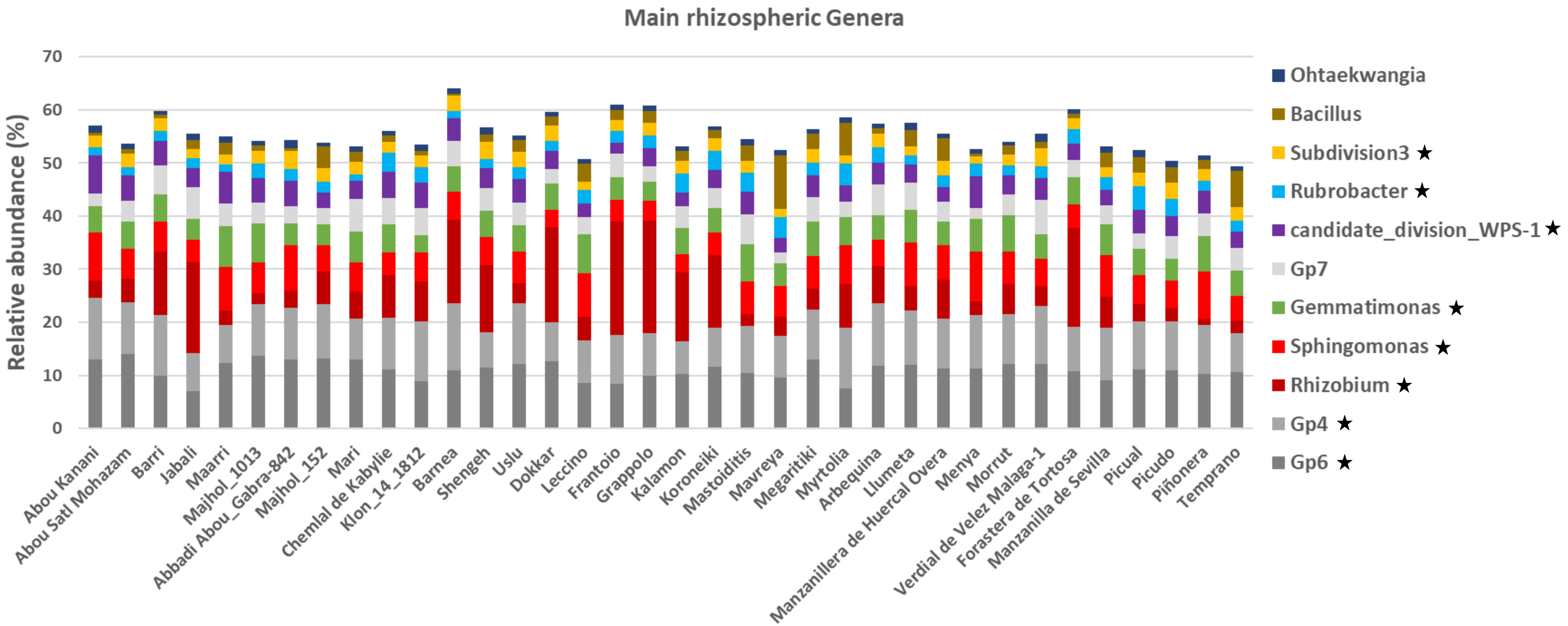

Figure S5. Statistically significant fungal endophytic (a) and rhizosphere (b) genera by cultivar and the main fungal genera in the endosphere (c) and the rhizosphere (d).

a)

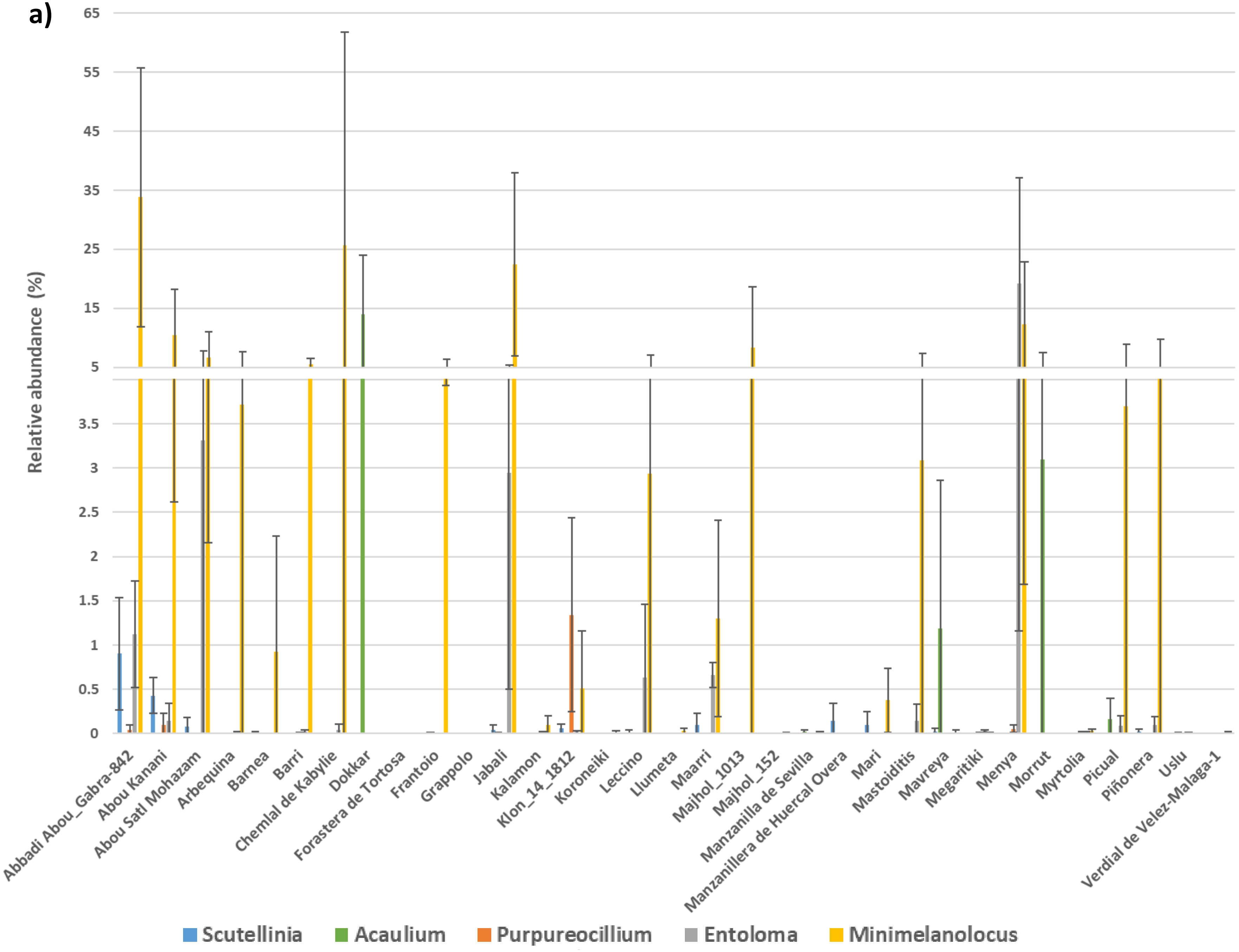

Figure S5. Statistically significant fungal endophytic (a) and rhizosphere (b) genera by cultivar and the main fungal genera in the endosphere (c) and the rhizosphere (d).

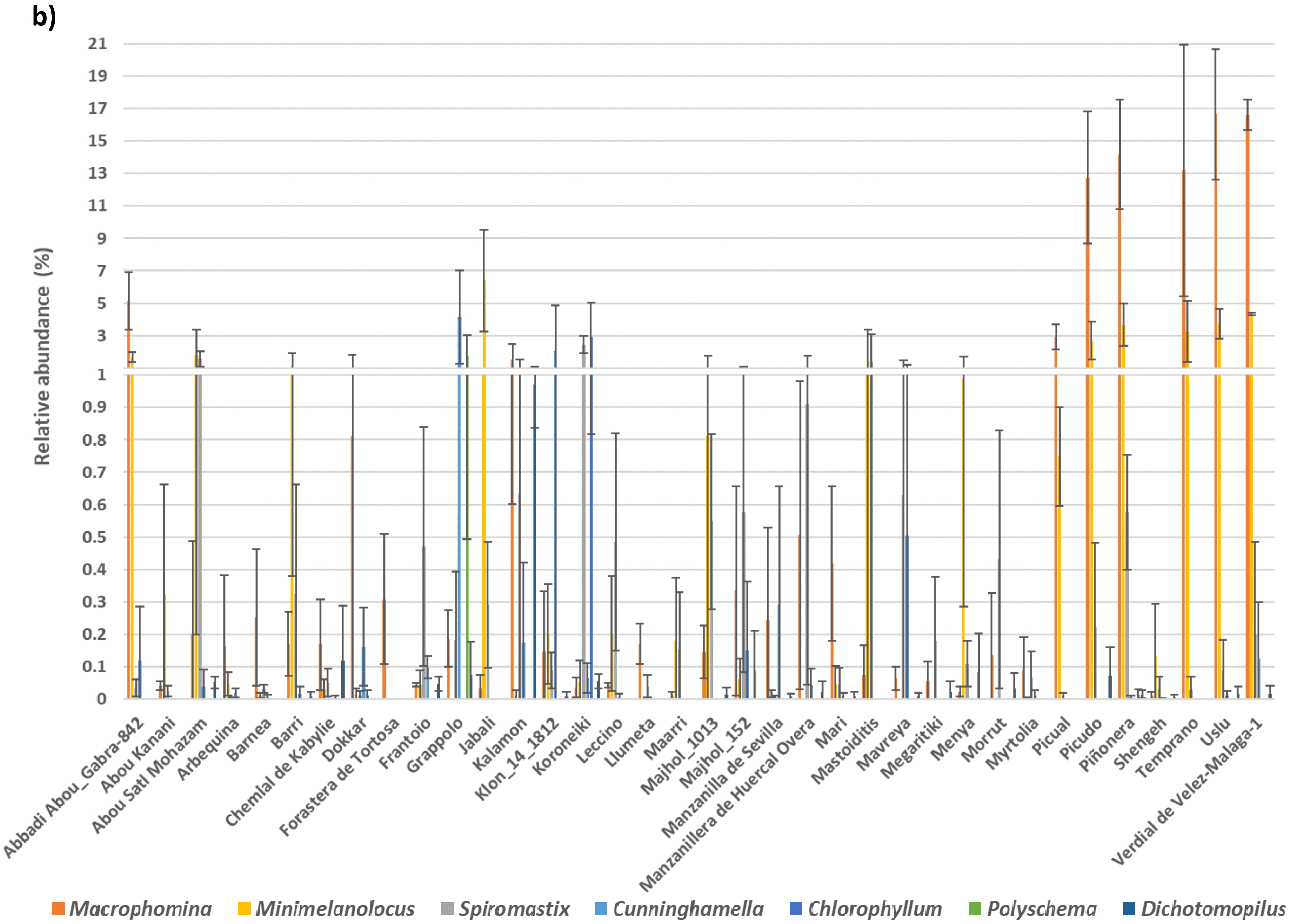

Figure S5. Statistically significant fungal endophytic (a) and rhizosphere (b) genera by cultivar and the main fungal genera in the endosphere (c) and the rhizosphere (d).

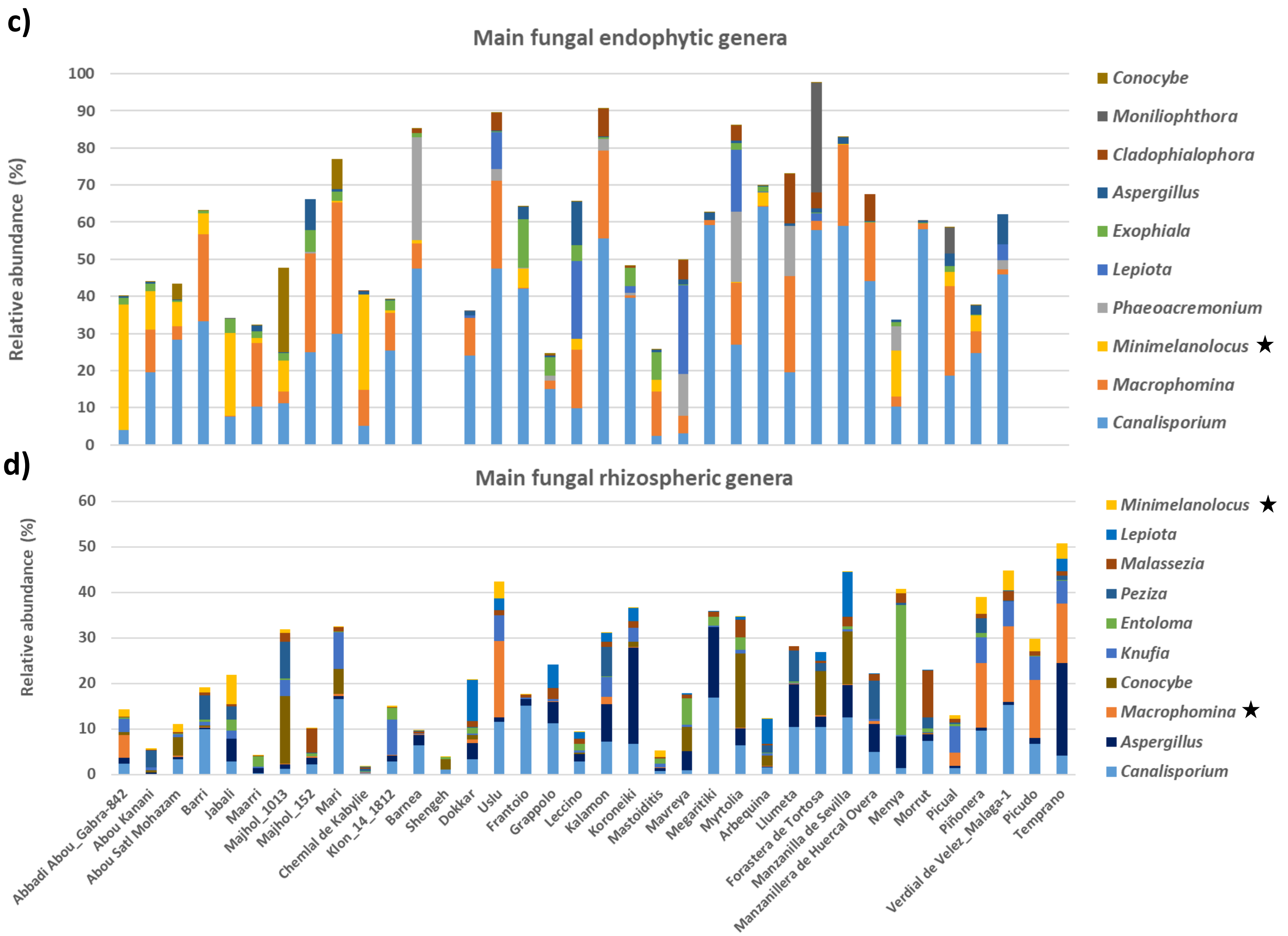

Table S1. Bacterial alpha diversity indices of each sample in both compartments.

| Samples | Sequences | OTUs | Chao1 | se.chao1 | ACE | se.ACE | Shannon | Simpson | InvSimpson | Fisher | Singletons | Good's coverage |
|---|---|---|---|---|---|---|---|---|---|---|---|---|
| E02A | 5090 | 453 | 637,078125 | 39,88877096 | 616,0401222 | 12,80322634 | 4,870018219 | 0,984310621 | 63,73738567 | 120,1785762 | 154 | 96,97% |
| E02B | 10314 | 431 | 566,3442623 | 31,67249555 | 566,1292989 | 12,15425684 | 4,424786048 | 0,970870738 | 34,32974025 | 90,92912408 | 129 | 98,75% |
| E02C | 3360 | 289 | 474,1086957 | 44,36303871 | 485,5996555 | 13,02017304 | 3,681299607 | 0,921691468 | 12,77000063 | 75,76681016 | 131 | 96,10% |
| E03A | 16069 | 436 | 634,9166667 | 43,30246011 | 633,2318942 | 13,71315259 | 3,528431571 | 0,885703135 | 8,749146382 | 82,65182363 | 155 | 99,04% |
| E03B | 5969 | 305 | 489,5238095 | 45,54356194 | 459,5031986 | 11,47469417 | 3,185711556 | 0,83084118 | 5,911604255 | 67,98328116 | 125 | 97,91% |
| E03C | 8619 | 412 | 602,7843137 | 44,02161616 | 580,6886462 | 12,9068105 | 4,423818283 | 0,972849508 | 36,83174453 | 90,13755339 | 140 | 98,38% |
| E04A | 6125 | 328 | 481,3571429 | 39,1466624 | 454,7504273 | 11,2356302 | 3,372173673 | 0,863092165 | 7,304183866 | 74,09368583 | 114 | 98,14% |
| E04B | 5308 | 333 | 549,1960784 | 48,76401837 | 561,9688338 | 13,58196519 | 3,501904351 | 0,887267175 | 8,870530868 | 78,82662615 | 149 | 97,19% |
| E04C | 2061 | 284 | 458 | 44,43359089 | 437,5713912 | 11,16242973 | 4,672640301 | 0,979915112 | 49,78867725 | 89,25701018 | 117 | 94,32% |
| E06A | 15999 | 409 | 553,5581395 | 37,04399089 | 514,8795545 | 11,39108937 | 4,073134917 | 0,96007985 | 25,05000606 | 76,47617404 | 112 | 99,30% |
| E06B | 39668 | 515 | 642,1694915 | 30,44423998 | 643,3122587 | 13,04589776 | 4,336299967 | 0,973727165 | 38,06212712 | 83,53407004 | 123 | 99,69% |
| E06C | 12487 | 457 | 662,5 | 48,3379135 | 605,0799424 | 12,65245965 | 4,227764566 | 0,959864998 | 24,91590745 | 93,15957766 | 138 | 98,89% |
| E07A | 6484 | 481 | 657,5538462 | 38,39855051 | 635,5195855 | 12,89613407 | 4,877132881 | 0,982918186 | 58,54179164 | 120,0156351 | 152 | 97,66% |
| E07B | 3081 | 276 | 462,1538462 | 46,9782657 | 437,2549624 | 11,42613115 | 4,063548343 | 0,960344 | 25,21686497 | 73,38914797 | 121 | 96,07% |
| E07C | 21728 | 445 | 661,6511628 | 51,60730715 | 594,2606433 | 12,4996234 | 3,926878427 | 0,946679964 | 18,75467606 | 79,2114424 | 137 | 99,37% |
| E08A | 4502 | 263 | 397,75 | 36,96206855 | 388,2044087 | 10,59494296 | 3,864427356 | 0,947671414 | 19,11001361 | 60,93794553 | 99 | 97,80% |
| E08B | 63565 | 385 | 514,8181818 | 31,57731726 | 513,1343314 | 11,92324902 | 1,885290472 | 0,632978646 | 2,724637109 | 54,51569061 | 120 | 99,81% |
| E08C | 2207 | 195 | 303,9375 | 32,25016336 | 318,1115533 | 10,01633452 | 3,766541694 | 0,949891898 | 19,95685237 | 51,60167612 | 84 | 96,19% |
| E09A | 6008 | 331 | 481,9622642 | 36,00414709 | 505,2221111 | 12,707195 | 3,898825115 | 0,934632153 | 15,29804087 | 75,38689916 | 127 | 97,89% |
| E09B | 12598 | 299 | 536,2727273 | 55,24011373 | 548,1584849 | 14,37958467 | 1,892657972 | 0,577196727 | 2,365166176 | 54,97582578 | 145 | 98,85% |
| E09C | 12906 | 370 | 549,5588235 | 47,66859042 | 485,5040682 | 11,28650403 | 3,745694553 | 0,921728041 | 12,77596746 | 71,05079002 | 111 | 99,14% |
| E14A | 18187 | 413 | 486,8867925 | 20,5692475 | 489,6164751 | 11,03938834 | 4,18988102 | 0,960983048 | 25,62988509 | 75,19304229 | 89 | 99,51% |
| E14B | 11956 | 395 | 504,0188679 | 27,80880009 | 495,3325558 | 11,4148158 | 4,191425239 | 0,966482769 | 29,83539992 | 78,48859072 | 108 | 99,10% |
| E14C | 5914 | 268 | 349,4468085 | 22,90340065 | 365,6582283 | 9,86431913 | 3,388150712 | 0,885036841 | 8,6984388 | 57,78199212 | 88 | 98,51% |
| E16A | 8459 | 303 | 430,1875 | 32,33265675 | 445,8805947 | 11,3248311 | 3,446456069 | 0,922680404 | 12,933332 | 61,43186645 | 111 | 98,69% |
| E16B | 7340 | 237 | 336,8780488 | 27,93902171 | 353,4366757 | 10,41518582 | 2,927398426 | 0,884716347 | 8,674256671 | 46,829177 | 91 | 98,76% |
| E16C | 31756 | 415 | 560,4102564 | 38,36052179 | 543,9776753 | 12,12143246 | 4,311140053 | 0,972893515 | 36,89154033 | 67,39946383 | 107 | 99,66% |
| E17A | 12438 | 350 | 491,9148936 | 35,52178641 | 502,3133712 | 11,93152526 | 3,665784242 | 0,939510902 | 16,53190461 | 66,91600505 | 116 | 99,07% |
| E17B | 8465 | 346 | 457,3 | 28,74017592 | 448,7429473 | 10,83908346 | 3,881110581 | 0,946960575 | 18,85389958 | 72,57250834 | 106 | 98,75% |
| E17C | 77388 | 535 | 691,0222222 | 38,86027277 | 684,5920159 | 13,04808535 | 4,573451447 | 0,978503671 | 46,51957137 | 77,44643029 | 119 | 99,85% |
| E18A | 6983 | 164 | 279,5555556 | 40,62704154 | 245,6512967 | 8,269773018 | 2,557757999 | 0,842574884 | 6,352226542 | 30,08307762 | 65 | 99,07% |
| E18B | 26481 | 339 | 441,375 | 28,69895677 | 423,8185189 | 10,32508053 | 2,810290691 | 0,844037866 | 6,411812757 | 54,83775278 | 91 | 99,66% |
| E18C | 4512 | 273 | 346,0285714 | 22,8363388 | 337,0354719 | 9,092627407 | 4,071922742 | 0,940842937 | 16,90415236 | 63,92064131 | 72 | 98,40% |
| E19A | 6425 | 308 | 451,1333333 | 36,24176273 | 455,0131911 | 11,69855801 | 4,074461522 | 0,960192536 | 25,12091692 | 67,43751208 | 114 | 98,23% |
| E19B | 6562 | 314 | 479,4545455 | 44,97437064 | 440,0441659 | 11,12227123 | 4,099788516 | 0,960658613 | 25,41852407 | 68,71690613 | 105 | 98,40% |
| E19C | 34393 | 438 | 640,7380952 | 49,22121729 | 597,7811223 | 12,66185944 | 3,533942879 | 0,913052655 | 11,50121371 | 70,78138373 | 131 | 99,62% |
| E21A | 6610 | 375 | 482,0192308 | 27,55603678 | 482,0589846 | 11,33535284 | 4,421715539 | 0,97094738 | 34,42030298 | 86,14167822 | 106 | 98,40% |
| E21B | 2207 | 155 | 214,0357143 | 20,58181502 | 228,8803996 | 8,154941967 | 3,525593042 | 0,947080068 | 18,89647159 | 38,00091029 | 58 | 97,37% |
| E21C | 32193 | 387 | 515,2692308 | 31,79390245 | 522,1220723 | 11,89417811 | 3,348940095 | 0,906064995 | 10,6456587 | 61,85425765 | 116 | 99,64% |
| E23A | 18907 | 462 | 595,2459016 | 31,28447405 | 586,4138886 | 12,10850465 | 3,061485413 | 0,776129353 | 4,466865187 | 85,50437813 | 128 | 99,32% |
| E23B | 12240 | 419 | 532 | 28,0192695 | 527,4687079 | 11,55000516 | 4,077363965 | 0,943329142 | 17,64575353 | 83,99176215 | 114 | 99,07% |
| E23C | 16928 | 423 | 560,0425532 | 34,53858493 | 529,949694 | 11,63311752 | 3,468805238 | 0,864031906 | 7,354666628 | 78,68421971 | 114 | 99,33% |
| E24A | 37128 | 436 | 504,3529412 | 19,57877338 | 502,5828358 | 11,13370492 | 3,497672227 | 0,906418958 | 10,68592499 | 69,37786646 | 84 | 99,77% |
| E24B | 5898 | 278 | 506,6 | 57,77452986 | 486,1370036 | 12,73581179 | 3,316229454 | 0,906838833 | 10,73408621 | 60,58590267 | 127 | 97,85% |
| E24C | 4980 | 210 | 299,1463415 | 25,53835497 | 327,8662158 | 10,60733953 | 2,914269881 | 0,879527991 | 8,30068339 | 44,41078748 | 86 | 98,27% |
| E25A | 4414 | 311 | 422,0566038 | 28,2163103 | 441,9265888 | 11,40980167 | 4,111695786 | 0,955019443 | 22,23182796 | 76,32502085 | 109 | 97,53% |
| E25B | 2815 | 214 | 341,96875 | 36,76255081 | 356,5398501 | 10,98060108 | 3,699761532 | 0,930423606 | 14,37269085 | 53,82370413 | 91 | 96,77% |
| E25C | 7478 | 304 | 426,9361702 | 31,66411802 | 438,4133858 | 11,28876018 | 3,609838039 | 0,925107801 | 13,35252559 | 63,67225038 | 108 | 98,56% |
| E27A | 3828 | 278 | 411,9 | 35,59730756 | 407,4930444 | 10,71984672 | 3,765073253 | 0,920848852 | 12,6340555 | 68,88991254 | 104 | 97,28% |
| E27B | 5877 | 264 | 359,2325581 | 26,53865108 | 366,1748459 | 9,909924739 | 3,527981342 | 0,930570079 | 14,4030122 | 56,78452113 | 91 | 98,45% |
| E27C | 3841 | 312 | 424,8139535 | 30,35384645 | 404,9077551 | 10,21021419 | 4,469820279 | 0,975400658 | 40,6514944 | 80,2177715 | 99 | 97,42% |
| E28A | 17834 | 520 | 594,8915663 | 18,54102343 | 611,1002598 | 12,20428067 | 4,439567526 | 0,970542191 | 33,94685595 | 100,2555533 | 112 | 99,37% |
| E28B | 4721 | 303 | 417,4666667 | 30,29952612 | 421,4473442 | 10,90278662 | 4,225231658 | 0,96447673 | 28,15056136 | 72,22614611 | 102 | 97,84% |
| E28C | 67698 | 798 | 993,6639344 | 34,77586934 | 1025,615117 | 16,69161301 | 4,099715864 | 0,95510507 | 22,27423011 | 127,0713014 | 219 | 99,68% |
| E29A | 3933 | 209 | 286,027027 | 23,44065452 | 303,8509376 | 9,434708527 | 3,178469551 | 0,825646642 | 5,735478867 | 47,10789571 | 76 | 98,07% |
| E29B | 9041 | 341 | 474,7837838 | 36,4357554 | 433,0227975 | 10,34247407 | 3,879191243 | 0,951369654 | 20,56329174 | 70,04851789 | 100 | 98,89% |
| E29C | 8428 | 293 | 392,8780488 | 27,93911691 | 385,6299948 | 10,13191226 | 3,7202342 | 0,940094818 | 16,69304671 | 58,9606538 | 91 | 98,92% |
| E30A | 6040 | 269 | 391,5263158 | 33,65553231 | 382,9025102 | 10,33904529 | 3,377193792 | 0,913793913 | 11,60010887 | 57,72571685 | 97 | 98,39% |
| E30B | 50587 | 560 | 715,7735849 | 36,92227589 | 701,3946792 | 13,44265898 | 4,195201795 | 0,943349523 | 17,65210209 | 88,12787355 | 129 | 99,74% |
| E30C | 17827 | 485 | 613,2692308 | 31,79396503 | 594,8113983 | 12,27617618 | 4,251802368 | 0,933889133 | 15,12610632 | 91,99805398 | 116 | 99,35% |
| E35A | 9093 | 233 | 319,0588235 | 26,21544718 | 326,914765 | 9,824018007 | 2,61746118 | 0,798953817 | 4,97398152 | 43,59048385 | 77 | 99,15% |
| E35B | 5550 | 184 | 309,125 | 39,54090862 | 296,3338392 | 9,534514531 | 2,821970768 | 0,860560734 | 7,171581052 | 36,59312004 | 78 | 98,59% |
| E35C | 4404 | 147 | 241,55 | 33,24562342 | 243,6268189 | 9,045837428 | 2,765595009 | 0,85806438 | 7,045447809 | 29,28364677 | 62 | 98,59% |
| E36A | 14233 | 358 | 498 | 34,28676632 | 498,6540109 | 12,00402628 | 2,964031965 | 0,824007246 | 5,682052112 | 66,69281086 | 120 | 99,16% |
| E36B | 2235 | 126 | 252,4285714 | 47,64304186 | 231,2280479 | 8,836873452 | 2,732628639 | 0,828546061 | 5,832470276 | 28,89022587 | 60 | 97,32% |
| E36C | 2854 | 140 | 204,1666667 | 22,97763477 | 216,9792168 | 8,298927765 | 2,588442396 | 0,796455042 | 4,912919542 | 30,84996598 | 56 | 98,04% |
| R01A | 47200 | 1873 | 1995,415385 | 21,76774355 | 1982,749023 | 21,68449002 | 6,207607875 | 0,993727018 | 159,4138188 | 389,829471 | 219 | 99,54% |
| R01B | 28071 | 1642 | 1788,768116 | 24,50686268 | 1791,213563 | 20,4652117 | 6,257293892 | 0,994617291 | 185,7800441 | 380,6034257 | 247 | 99,12% |
| R01C | 42465 | 1731 | 1884,151685 | 26,20787325 | 1858,931404 | 20,98131084 | 6,050291155 | 0,992920406 | 141,2510438 | 362,8127504 | 234 | 99,45% |
| R02A | 42726 | 2015 | 2129,4 | 20,84704141 | 2111,371259 | 22,44466889 | 6,484470541 | 0,995102999 | 204,2066055 | 439,2063553 | 209 | 99,51% |
| R02B | 34476 | 1915 | 2052,4 | 23,82112471 | 2027,573169 | 21,72439853 | 6,541256915 | 0,996392647 | 277,2115867 | 437,1892892 | 229 | 99,34% |
| R02C | 41872 | 1884 | 2031,819767 | 25,74057894 | 1996,957185 | 21,74697762 | 6,281959693 | 0,992833753 | 139,543051 | 405,4142189 | 226 | 99,46% |
| R03A | 47001 | 1754 | 1907,377778 | 28,10492816 | 1862,478787 | 21,26874804 | 6,316027831 | 0,995400532 | 217,4164526 | 359,3307231 | 204 | 99,57% |
| R03B | 40273 | 1701 | 1846,459459 | 26,38779798 | 1814,450412 | 20,98950778 | 6,2571313 | 0,9943309 | 176,3948548 | 359,8812812 | 208 | 99,48% |
| R03C | 25953 | 1652 | 1823,861386 | 27,68716725 | 1805,476105 | 20,10393187 | 6,176181715 | 0,992727706 | 137,5082011 | 392,7925696 | 264 | 98,98% |
| R04A | 17295 | 1631 | 1950,714286 | 43,43766749 | 1888,709063 | 20,12618031 | 6,23508787 | 0,992689767 | 136,7945546 | 441,6660653 | 373 | 97,84% |
| R04B | 46994 | 1973 | 2103,679558 | 23,22270697 | 2075,190263 | 22,13789806 | 6,24147656 | 0,992410396 | 131,759172 | 416,7650756 | 218 | 99,54% |
| R04C | 42655 | 1911 | 2019,341969 | 19,97505211 | 2008,192358 | 21,69686083 | 6,023675566 | 0,98593182 | 71,08239843 | 410,7462384 | 205 | 99,52% |
| R05A | 43289 | 1888 | 2046,80102 | 26,30421809 | 2013,874693 | 21,52002399 | 5,868772233 | 0,976753128 | 43,01654071 | 402,8778344 | 250 | 99,42% |
| R05B | 51449 | 1900 | 2033,115183 | 23,2469197 | 2014,619181 | 21,8349948 | 5,654680548 | 0,966562013 | 29,90610691 | 388,204148 | 226 | 99,56% |
| R05C | 38544 | 1858 | 1980,044776 | 21,57249072 | 1965,159434 | 21,11703138 | 5,860666021 | 0,972024118 | 35,7450746 | 407,4403768 | 222 | 99,42% |
| R06A | 44232 | 1991 | 2112,916667 | 22,1021937 | 2089,234553 | 22,33565341 | 6,193830162 | 0,985698357 | 69,92203864 | 428,4867671 | 210 | 99,53% |
| R06B | 47777 | 1808 | 1951,78125 | 25,66307081 | 1920,747087 | 21,56725259 | 5,97087714 | 0,98132044 | 53,53445173 | 371,7168399 | 215 | 99,55% |
| R06C | 36746 | 1908 | 2082,931034 | 28,01955685 | 2053,201409 | 21,84518812 | 6,039140639 | 0,979759772 | 49,4065592 | 427,2247007 | 267 | 99,27% |
| R07A | 36411 | 1821 | 1929,780612 | 19,96542501 | 1918,40165 | 21,0890532 | 5,936487974 | 0,972917427 | 36,92411345 | 403,4474026 | 207 | 99,43% |

| | | | | | | | | | | | | |
|---|---|---|---|---|---|---|---|---|---|---|---|---|
| R07B | 45268 | 1844 | 1972,439759 | 23,39969638 | 1951,651944 | 21,78886094 | 6,400175972 | 0,99503053 | 201,2286885 | 386,4280635 | 207 | 99,54% |
| R07C | 40281 | 1801 | 1946,285714 | 25,30743446 | 1918,512633 | 21,2867692 | 6,195551277 | 0,992906603 | 140,9761766 | 386,8917119 | 226 | 99,44% |
| R08A | 38945 | 1814 | 1947,155251 | 22,53942531 | 1947,84599 | 21,41540585 | 6,18298195 | 0,992984563 | 142,5427909 | 394,0479911 | 242 | 99,38% |
| R08B | 37848 | 1756 | 1910,570652 | 26,1775004 | 1882,665468 | 20,8614598 | 5,872697869 | 0,97953416 | 48,86190913 | 381,0393366 | 239 | 99,37% |
| R08C | 41915 | 1916 | 2091,127389 | 29,8914427 | 2030,364346 | 21,83033843 | 6,263150431 | 0,992134478 | 127,137151 | 414,0748522 | 235 | 99,44% |
| R09A | 33021 | 1807 | 2033,091954 | 35,33318379 | 1957,961286 | 20,90493165 | 5,99009375 | 0,980844755 | 52,20502375 | 410,7491644 | 281 | 99,15% |
| R09B | 27439 | 1748 | 1967,945946 | 32,51281788 | 1943,160887 | 20,86690222 | 5,974694737 | 0,980582043 | 51,49872354 | 415,7209614 | 313 | 98,86% |
| R09C | 28559 | 1695 | 1856,674312 | 25,99175115 | 1846,73426 | 20,32939835 | 6,023400684 | 0,982789626 | 58,10448844 | 394,589491 | 266 | 99,07% |
| R10A | 35284 | 1756 | 1961,516129 | 32,30331575 | 1915,370364 | 21,02933976 | 5,71749616 | 0,963450337 | 27,36003364 | 388,5116996 | 277 | 99,21% |
| R10B | 30683 | 1543 | 1735,714286 | 29,86310978 | 1735,864259 | 20,11089432 | 5,690912451 | 0,973817461 | 38,19339358 | 342,3847545 | 285 | 99,07% |
| R10C | 38204 | 1856 | 1996,310044 | 23,17670515 | 1992,733504 | 21,38448961 | 5,697758881 | 0,958270696 | 23,96397533 | 407,8817963 | 254 | 99,34% |
| R11A | 32817 | 1752 | 1905,261468 | 24,99374916 | 1896,554426 | 20,50421699 | 5,708859635 | 0,964459618 | 28,13700799 | 395,4201367 | 259 | 99,21% |
| R11B | 40991 | 1921 | 2070,727273 | 25,1277614 | 2041,618923 | 21,63578206 | 5,838595402 | 0,965230466 | 28,76081139 | 417,9909216 | 244 | 99,40% |
| R11C | 39067 | 1879 | 2012,171429 | 22,75538326 | 1998,269437 | 21,44121548 | 5,701912888 | 0,959141205 | 24,47453467 | 411,7900094 | 237 | 99,39% |
| R12A | 39927 | 1872 | 2068,281407 | 30,67993761 | 2026,368099 | 21,54007698 | 5,681284579 | 0,960639387 | 25,40610821 | 407,3805818 | 280 | 99,30% |
| R12B | 47320 | 1732 | 1865,876923 | 23,23269937 | 1852,401414 | 20,71801565 | 5,342130224 | 0,943607349 | 17,73280701 | 353,0778685 | 229 | 99,52% |
| R12C | 46766 | 1884 | 2009,014493 | 21,80700033 | 2000,205944 | 21,67906451 | 5,57757591 | 0,950476008 | 20,19223335 | 393,6651112 | 228 | 99,51% |
| R13A | 47812 | 1998 | 2090,871429 | 17,57491014 | 2083,048619 | 21,77385135 | 5,471885659 | 0,934439796 | 15,25315577 | 421,5252112 | 198 | 99,59% |
| R13B | 38697 | 1916 | 2105,445714 | 30,85372649 | 2045,689021 | 21,61368067 | 5,898398905 | 0,97152589 | 35,11962219 | 423,3029932 | 258 | 99,33% |
| R13C | 35960 | 1484 | 1645,119565 | 26,99545498 | 1637,4891 | 19,56120171 | 5,275581974 | 0,947672261 | 19,11032299 | 312,0507308 | 244 | 99,32% |
| R14A | 41401 | 1748 | 1868,678571 | 21,51862105 | 1865,233938 | 21,03577157 | 5,654181216 | 0,955236298 | 22,33952862 | 369,789003 | 218 | 99,47% |
| R14B | 45113 | 1692 | 1826,73494 | 24,24000081 | 1807,020024 | 20,8571932 | 5,666372093 | 0,973247344 | 37,37946584 | 347,0730477 | 212 | 99,53% |
| R14C | 37875 | 1901 | 2082,759162 | 29,2621625 | 2039,362253 | 21,71249135 | 5,877256676 | 0,966671505 | 30,00435503 | 421,5944764 | 264 | 99,30% |
| R15A | 50499 | 1880 | 2033,12426 | 26,53691094 | 1994,085222 | 21,77201136 | 5,900161209 | 0,974005115 | 38,46910711 | 384,9066528 | 228 | 99,55% |
| R15B | 44422 | 1736 | 1858,415385 | 21,76774235 | 1854,609687 | 21,07026722 | 6,059432806 | 0,986216865 | 72,55243566 | 359,8839386 | 219 | 99,51% |
| R15C | 38354 | 1814 | 2000,653631 | 30,33726686 | 1952,504837 | 21,18302587 | 5,862463392 | 0,979421205 | 48,59371135 | 395,7065036 | 259 | 99,32% |
| R16A | 40412 | 1822 | 2007,853107 | 30,32505729 | 1960,043643 | 21,4062628 | 5,820320634 | 0,965881126 | 29,30929078 | 392,2880724 | 257 | 99,36% |
| R16B | 22658 | 1691 | 1892,253846 | 29,29202209 | 1900,531073 | 20,19009306 | 5,991029845 | 0,985598978 | 69,43951768 | 422,7537498 | 324 | 98,57% |
| R16C | 38491 | 1917 | 2038,542735 | 20,80105873 | 2039,02499 | 21,58373358 | 6,001798269 | 0,980674507 | 51,74512236 | 424,2191813 | 239 | 99,38% |
| R17A | 39457 | 1703 | 1862,780899 | 27,04582868 | 1830,041031 | 20,56089032 | 6,013811128 | 0,992148456 | 127,3634774 | 362,3869481 | 239 | 99,39% |
| R17B | 28070 | 1687 | 1840,368984 | 25,92600277 | 1813,980682 | 20,18811271 | 6,238976197 | 0,993714825 | 159,1045558 | 394,2011353 | 240 | 99,14% |
| R17C | 38694 | 1895 | 2048,606218 | 25,76081442 | 2014,048359 | 21,36485296 | 6,204307119 | 0,992677492 | 136,5652402 | 417,3865186 | 244 | 99,37% |
| R18A | 45617 | 1678 | 1872,188679 | 32,21918748 | 1831,960013 | 21,09223216 | 6,082759762 | 0,992526768 | 133,8109078 | 342,5022688 | 249 | 99,45% |
| R18B | 30033 | 1630 | 1828,784884 | 32,13706142 | 1787,313634 | 20,35968265 | 6,181998818 | 0,99436228 | 177,3766639 | 369,6336002 | 262 | 99,13% |
| R18C | 59509 | 1910 | 2049,47619 | 25,61512044 | 2006,565338 | 21,99074224 | 6,140630794 | 0,992189123 | 128,0265985 | 376,8482683 | 203 | 99,66% |
| R19A | 33182 | 1819 | 1941,160804 | 21,63580269 | 1926,080531 | 20,93329905 | 6,363278554 | 0,995181807 | 207,5466846 | 413,6870991 | 221 | 99,33% |
| R19B | 42106 | 1837 | 1964,111111 | 23,05996988 | 1940,85183 | 21,54643446 | 6,370017214 | 0,995541603 | 224,2958615 | 392,0290711 | 209 | 99,50% |
| R19C | 38940 | 1696 | 1850,466292 | 26,37457643 | 1826,120532 | 20,77880005 | 6,236953612 | 0,994758668 | 190,7911816 | 361,7744318 | 235 | 99,40% |
| R20A | 49011 | 1883 | 2006,034483 | 21,65089146 | 1998,199821 | 21,84334936 | 6,163677017 | 0,992465661 | 132,7256436 | 388,64564 | 224 | 99,54% |
| R20B | 51268 | 1951 | 2081,034884 | 23,41631632 | 2053,549093 | 22,20642731 | 6,264873713 | 0,993111769 | 145,1751566 | 401,6934599 | 212 | 99,59% |
| R20C | 56183 | 1859 | 2006,9 | 27,09342385 | 1954,432016 | 21,64124926 | 6,152909695 | 0,992056072 | 125,8823091 | 369,5319588 | 204 | 99,64% |
| R21A | 50039 | 1929 | 2024,761628 | 18,74637495 | 2010,387527 | 21,98671987 | 6,351460898 | 0,994600864 | 185,2148174 | 398,4865106 | 182 | 99,64% |
| R21B | 48322 | 1715 | 1874,071856 | 27,38160555 | 1845,375156 | 21,03411199 | 5,81330851 | 0,981863367 | 55,13702637 | 346,9025365 | 231 | 99,52% |
| R21C | 38834 | 1877 | 2046,262376 | 27,37641111 | 2020,084562 | 21,68529404 | 6,242929239 | 0,99233243 | 130,4194109 | 411,9126592 | 262 | 99,33% |
| R22A | 46857 | 1980 | 2126,892216 | 25,80435444 | 2085,441105 | 22,26694254 | 6,216934685 | 0,991066216 | 111,9346552 | 418,9607192 | 222 | 99,53% |
| R22B | 49797 | 1990 | 2086,390533 | 18,9080145 | 2068,515157 | 22,32299395 | 6,444604722 | 0,995469837 | 220,7425869 | 414,9399899 | 181 | 99,64% |
| R22C | 39078 | 1874 | 2039,138122 | 27,60492056 | 2004,952993 | 21,86699865 | 6,427415856 | 0,994968502 | 198,7479855 | 410,359484 | 245 | 99,37% |
| R23A | 78913 | 2051 | 2131,930233 | 17,69879173 | 2113,391578 | 22,52305896 | 6,335681718 | 0,994063878 | 168,4601468 | 384,959824 | 145 | 99,82% |
| R23B | 40825 | 1902 | 1998,032432 | 18,48868096 | 1985,388189 | 21,59833868 | 6,418317921 | 0,995704221 | 232,7866698 | 413,1896187 | 189 | 99,54% |
| R23C | 39686 | 1877 | 1998,719577 | 21,83170707 | 1977,289105 | 21,36326063 | 6,294391326 | 0,994515708 | 182,3389527 | 409,449427 | 215 | 99,46% |
| R24A | 43656 | 1660 | 1822,958115 | 26,97880308 | 1812,679811 | 20,75427292 | 5,633281959 | 0,97993333 | 49,83387811 | 341,7091177 | 250 | 99,43% |
| R24B | 41088 | 1889 | 2035,283582 | 24,61538694 | 2013,458542 | 21,66487826 | 6,173697495 | 0,992393201 | 131,4613484 | 408,8783759 | 243 | 99,41% |
| R24C | 54593 | 2042 | 2129,092025 | 17,71827339 | 2110,575796 | 22,54262473 | 6,473606708 | 0,99597661 | 248,5466454 | 418,5764926 | 169 | 99,69% |
| R25A | 45925 | 1964 | 2079,28866 | 20,86672311 | 2068,013994 | 22,30645829 | 6,484926592 | 0,996278774 | 268,7286288 | 416,9007226 | 212 | 99,54% |
| R25B | 41720 | 2088 | 2200,160221 | 20,77561084 | 2176,916076 | 22,83897941 | 6,658397909 | 0,996743976 | 307,1230392 | 462,6943029 | 202 | 99,52% |
| R25C | 34251 | 1872 | 2006,56701 | 23,34774819 | 1980,119086 | 21,18536674 | 6,358700262 | 0,995049578 | 202,0029987 | 425,3725121 | 229 | 99,33% |
| R26A | 45942 | 1845 | 1949,923913 | 19,72575148 | 1939,942552 | 21,4779885 | 6,284492457 | 0,995001734 | 200,0694016 | 385,1997734 | 197 | 99,57% |
| R26B | 48109 | 1424 | 1601,456954 | 30,49464999 | 1581,959725 | 19,62516776 | 5,746587759 | 0,98733216 | 78,94005378 | 275,5288486 | 232 | 99,52% |
| R26C | 30887 | 1772 | 1951,161943 | 27,21950382 | 1950,883313 | 20,81245415 | 5,960724389 | 0,98873446 | 88,76627652 | 408,3883199 | 298 | 99,04% |
| R27A | 26555 | 1689 | 1869,311321 | 28,35733533 | 1848,041065 | 20,10793618 | 6,20908147 | 0,994640581 | 186,587385 | 401,4940904 | 277 | 98,96% |
| R27B | 41653 | 1838 | 1992,508287 | 26,27352571 | 1956,800391 | 21,32440755 | 6,184658503 | 0,993545972 | 154,9419922 | 393,4413954 | 237 | 99,43% |
| R27C | 42324 | 1969 | 2066,20339 | 18,8301871 | 2046,08759 | 21,85615329 | 6,329713454 | 0,993666463 | 157,889655 | 427,5759991 | 186 | 99,56% |
| R28A | 38579 | 1712 | 1871,606061 | 27,53219584 | 1836,585798 | 20,88878543 | 6,239931893 | 0,994895062 | 195,8887791 | 367,0280332 | 230 | 99,40% |
| R28B | 51493 | 1829 | 1961,236842 | 24,41835194 | 1929,374512 | 21,61211424 | 6,254694994 | 0,994263863 | 174,3333638 | 370,036096 | 201 | 99,61% |
| R28C | 62077 | 1983 | 2037,721519 | 12,84477362 | 2037,639156 | 22,29504924 | 6,468318816 | 0,995866091 | 241,9017973 | 390,7996335 | 132 | 99,79% |
| R29A | 45873 | 1863 | 2011,505495 | 25,48048115 | 1989,565035 | 21,86191663 | 6,304792788 | 0,994061954 | 168,4055662 | 390,1001762 | 233 | 99,49% |
| R29B | 57876 | 1835 | 1953,452381 | 21,98870834 | 1932,918236 | 21,60283273 | 6,102282993 | 0,991784129 | 121,7156372 | 360,9738964 | 200 | 99,65% |
| R29C | 34373 | 1816 | 1971,975728 | 25,65426493 | 1954,464987 | 21,29856205 | 6,275857192 | 0,992756103 | 138,0472412 | 408,6398853 | 254 | 99,26% |
| R30A | 41245 | 1800 | 1958,989362 | 26,59167799 | 1936,148684 | 21,41223205 | 6,026609332 | 0,988409744 | 86,27936809 | 384,1652673 | 245 | 99,41% |
| R30B | 45724 | 1907 | 2016,340659 | 20,37102418 | 1999,600982 | 21,72362571 | 6,165339469 | 0,99079441 | 108,6296442 | 402,1180009 | 200 | 99,56% |
| R30C | 58915 | 1821 | 1999,903226 | 30,47709773 | 1943,831369 | 21,48500453 | 5,667757678 | 0,982706902 | 57,82653703 | 356,0193376 | 236 | 99,60% |
| R31A | 35816 | 1773 | 1910,171123 | 23,88050852 | 1891,2797 | 20,94271434 | 6,213039111 | 0,993604008 | 156,3479143 | 391,6880898 | 227 | 99,37% |
| R31B | 62754 | 1875 | 1999,22293 | 23,13467692 | 1972,971584 | 21,87982808 | 6,152823206 | 0,993195803 | 146,9681181 | 363,6056873 | 198 | 99,68% |
| R31C | 51630 | 1885 | 1998,460606 | 21,39656781 | 1982,080435 | 21,94652125 | 6,400629323 | 0,995850212 | 240,9761877 | 384,0224094 | 194 | 99,62% |
| R32A | 50519 | 1836 | 1979,78125 | 25,66307116 | 1947,37066 | 21,66709893 | 6,247090682 | 0,994619074 | 185,8416119 | 373,6056963 | 215 | 99,57% |
| R32B | 62337 | 1909 | 2025,117647 | 22,14831439 | 1997,13741 | 22,01208662 | 6,205084187 | 0,994460087 | 180,5082467 | 372,3919152 | 189 | 99,70% |
| R32C | 15565 | 1536 | 1732,887097 | 29,14698345 | 1745,330248 | 19,27221314 | 6,37483643 | 0,995983935 | 248,9999424 | 422,838385 | 313 | 97,99% |
| R33A | 43948 | 1926 | 2056,442623 | 23,13270262 | 2034,412566 | 22,06022446 | 6,344615419 | 0,994483284 | 181,2672714 | 411,5183218 | 219 | 99,50% |
| R33B | 60426 | 2003 | 2075,007092 | 15,9554921 | 2060,187387 | 22,3487017 | 6,471432789 | 0,995542722 | 224,3521959 | 398,3329238 | 143 | 99,76% |
| R33C | 44465 | 2054 | 2171,487952 | 21,91834007 | 2141,682854 | 22,69924127 | 6,636651761 | 0,996736891 | 306,4562053 | 445,1705378 | 198 | 99,55% |
| R34A | 42324 | 1744 | 1882,550725 | 23,49715099 | 1872,967887 | 20,58682957 | 5,813269288 | 0,987259555 | 78,4901953 | 366,577211 | 240 | 99,43% |
| R34B | 45182 | 1742 | 1893,383562 | 24,74337342 | 1884,382345 | 20,67497038 | 5,33558812 | 0,967911991 | 31,16428977 | 359,8692848 | 258 | 99,43% |
| R34C | 36858 | 1718 | 1880,642857 | 26,77315113 | 1863,177019 | 20,92443153 | 6,243065032 | 0,995092151 | 203,7552673 | 373,2663059 | 253 | 99,31% |
| R35A | 61702 | 1925 | 2016,666667 | 18,26061246 | 2006,971759 | 22,09101619 | 6,27646549 | 0,995024837 | 200,9984202 | 377,2002486 | 176 | 99,71% |
| R35B | 46150 | 1948 | 2114,005291 | 27,42201292 | 2081,912704 | 22,29596714 | 6,267504973 | 0,993650656 | 157,4965821 | 412,0704243 | 251 | 99,46% |
| R35C | 39245 | 1739 | 1879,169399 | 24,38625064 | 1859,641641 | 21,01643077 | 6,246409801 | 0,993901808 | 163,9830214 | 372,6689433 | 227 | 99,42% |
| R36A | 45948 | 1648 | 1780,664671 | 23,93030578 | 1766,326675 | 20,69582115 | 5,999368361 | 0,991112737 | 112,5205807 | 334,2344419 | 211 | 99,54% |
| R36B | 48471 | 1830 | 1991,596591 | 27,34973105 | 1965,424763 | 21,78300448 | 6,230989822 | 0,994277283 | 174,7421836 | 376,0157383 | 239 | 99,51% |

| | | | | | | | | | | | | |
|---|---|---|---|---|---|---|---|---|---|---|---|---|
| R36C | 64551 | 1982 | 2063,50625 | 16,96521192 | 2053,686002 | 22,34148041 | 6,399680649 | 0,995347347 | 214,9311211 | 386,876602 | 162 | 99,75% |

Table S2. Fungal alpha diversity indices of each sample in both compartments.

| Samples | Sequences | OTUs | Chao1 | se.chao1 | ACE | se.ACE | Shannon | Simpson | InvSimpson | Fisher | Singletons | Good's coverage |
|---|---|---|---|---|---|---|---|---|---|---|---|---|
| E01A | 9072 | 36 | 43 | 7,103880368 | 42,25205761 | 2,894334868 | 1,404905714 | 0,566404294 | 2,306295901 | 4,767119031 | 7 | 99,92% |
| E01B | 4557 | 54 | 61 | 7,109794676 | 57,79596884 | 3,589133509 | 2,660301548 | 0,885503203 | 8,73386876 | 8,607380231 | 7 | 99,85% |
| E01C | 3311 | 31 | 49 | 16,09284288 | 44,23918367 | 3,453067629 | 2,28491552 | 0,873217151 | 7,887502203 | 4,73119575 | 9 | 99,73% |
| E02A | 23366 | 53 | 59,5 | 4,879918931 | 68,48604938 | 4,905280635 | 1,618362857 | 0,736385436 | 3,793417117 | 6,469564659 | 13 | 99,94% |
| E02B | 20489 | 39 | 43,66666667 | 4,488159692 | 46,34578186 | 3,338172281 | 1,774670167 | 0,77189143 | 4,383877387 | 4,647542615 | 8 | 99,96% |
| E02C | 2572 | 25 | 25,33333333 | 0,757727381 | 26,55555556 | 2,503264989 | 1,70045057 | 0,751567606 | 4,025239956 | 3,841365038 | 3 | 99,88% |
| E03A | 3104 | 41 | 51,5 | 10,53795728 | 47,13065417 | 3,365671429 | 2,522080609 | 0,863365113 | 7,31877501 | 6,672532315 | 7 | 99,77% |
| E03B | 2339 | 40 | 41,42857143 | 1,933181585 | 43,37680882 | 3,303142389 | 2,466147227 | 0,871433165 | 7,778055643 | 6,854619247 | 5 | 99,79% |
| E03C | 11929 | 57 | 79,66666667 | 14,90800127 | 84,62656585 | 4,961262038 | 1,983092269 | 0,740088317 | 3,847460761 | 7,768527784 | 17 | 99,86% |
| E04A | 6300 | 28 | 46,2 | 13,14186371 | 58,38666667 | 4,672635493 | 0,421852032 | 0,157831897 | 1,187411393 | 3,773068956 | 14 | 99,78% |
| E04B | 4263 | 27 | 36,16666667 | 7,372887864 | 47,26591315 | 4,114454238 | 0,465046198 | 0,182854735 | 1,223772618 | 3,85155595 | 11 | 99,74% |
| E04C | 5191 | 30 | 52 | 17,44172225 | 62,40645905 | 4,836684913 | 1,549106683 | 0,70072222 | 3,34137737 | 4,215409554 | 12 | 99,77% |
| E05A | 25786 | 17 | 35 | 16,06071071 | 57,98367347 | 2,978912984 | 1,105684856 | 0,628243363 | 2,689931798 | 1,773689932 | 9 | 99,97% |
| E05B | 3526 | 14 | 16 | 2,867441756 | 19,99412111 | 2,133140613 | 0,980991543 | 0,547868858 | 2,21174767 | 1,854021201 | 4 | 99,89% |
| E05C | 7152 | 27 | 32 | 5,522680509 | 33,44636678 | 3,052022716 | 1,444182949 | 0,645974484 | 2,824655157 | 3,548355392 | 6 | 99,92% |
| E06A | 17239 | 52 | 67,6 | 11,64038659 | 64,47034286 | 4,015795712 | 1,782348167 | 0,770090943 | 4,349545923 | 6,610134247 | 13 | 99,92% |
| E06B | 24337 | 37 | 44,2 | 6,43276905 | 48,33632653 | 3,455808129 | 1,969652606 | 0,813461022 | 5,360809894 | 4,27937532 | 9 | 99,96% |
| E06C | 12730 | 41 | 57,5 | 12,89124134 | 56,53136712 | 3,865409002 | 1,539450497 | 0,637373969 | 2,757661926 | 5,262074151 | 12 | 99,91% |
| E07A | 10622 | 37 | 40 | 3,415259873 | 40,3962449 | 2,834860858 | 1,285704671 | 0,503917967 | 2,015795641 | 4,804164512 | 6 | 99,94% |
| E07B | 16197 | 30 | 41,25 | 9,538690898 | 46,31001372 | 3,92270228 | 1,411812497 | 0,673985465 | 3,067347898 | 3,561882808 | 10 | 99,94% |
| E07C | 13001 | 38 | 43,14285714 | 4,648433142 | 48,83705882 | 3,627899188 | 1,310034956 | 0,62669144 | 2,678749184 | 4,808434842 | 9 | 99,93% |
| E08A | 1475 | 21 | 22 | 1,57754124 | 25,1776907 | 2,567939956 | 0,934792357 | 0,390248319 | 1,64001188 | 3,468123654 | 4 | 99,73% |
| E08B | 22253 | 23 | 30,2 | 6,428303722 | 34,45454545 | 2,228087266 | 1,153805602 | 0,61798284 | 2,617683457 | 2,532749427 | 9 | 99,96% |
| E08C | 2116 | 22 | 29 | 6,637147289 | 40,29250892 | 3,598660148 | 1,252332 | 0,548126883 | 2,213010604 | 3,42219904 | 8 | 99,62% |
| E10A | 19795 | 23 | 37 | 13,16475639 | 35,9122807 | 2,350502068 | 1,618981687 | 0,752309475 | 4,037296133 | 2,570002518 | 8 | 99,96% |
| E10B | 8219 | 18 | 63 | 30,1300093 | 50,22222222 | 3,324105194 | 1,407012903 | 0,73781109 | 3,814043856 | 2,186550383 | 10 | 99,88% |
| E10C | 895 | 18 | 21 | 3,410488025 | 29,15948276 | 3,647030877 | 1,013290297 | 0,454867201 | 1,834415399 | 3,191561631 | 6 | 99,33% |
| E11A | 13903 | 23 | 33,5 | 10,51367269 | 30,7549106 | 2,645147299 | 0,525599271 | 0,212921218 | 1,270520845 | 2,689883763 | 7 | 99,95% |
| E11B | 1669 | 17 | 17,5 | 1,024307741 | 19,00125 | 1,892275499 | 0,343516631 | 0,107914348 | 1,120968595 | 2,634487351 | 3 | 99,82% |
| E11C | 4668 | 27 | 32,6 | 5,338962469 | 37,39788977 | 3,1849299 | 0,869995886 | 0,384953454 | 1,625893206 | 3,79438613 | 8 | 99,83% |
| E12A | 5651 | 31 | 52 | 14,73381119 | 55,11605343 | 4,278937611 | 0,315473148 | 0,111061129 | 1,124936745 | 4,319181688 | 15 | 99,73% |
| E12B | 1680 | 24 | 26 | 2,580474017 | 29,07102273 | 2,816418317 | 1,579082918 | 0,670305414 | 3,033110163 | 3,966226853 | 5 | 99,70% |
| E12C | 24537 | 19 | 21 | 2,579036365 | 25,92689655 | 2,838930686 | 1,250345076 | 0,65513587 | 2,899692697 | 2,020268975 | 5 | 99,98% |
| E13A | 24591 | 39 | 44,25 | 5,366023167 | 47,29922665 | 3,36484958 | 1,386850156 | 0,627281644 | 2,682991012 | 4,535766635 | 7 | 99,97% |
| E13B | 4856 | 14 | 21,5 | 8,10280959 | 33,896 | 3,545272964 | 1,140499773 | 0,63645876 | 2,750719563 | 1,768043395 | 6 | 99,88% |
| E13C | 1184 | 17 | 24 | 7,087988666 | 25,89 | 2,064559468 | 1,009695862 | 0,426682912 | 1,744235469 | 2,812217029 | 7 | 99,41% |
| E14A | 3226 | 20 | 22 | 2,579376246 | 24,75530351 | 2,363103942 | 1,186772316 | 0,493686027 | 1,97505906 | 2,84290732 | 5 | 99,85% |
| E14B | 11377 | 37 | 59,5 | 19,27459413 | 51,23224271 | 3,42887944 | 2,135811715 | 0,837170262 | 6,141384313 | 4,755499928 | 10 | 99,91% |
| E14C | 9665 | 47 | 52 | 4,311307159 | 56,2924996 | 3,945542937 | 2,267316557 | 0,837336205 | 6,14764952 | 6,423414745 | 10 | 99,90% |
| E15A | 3431 | 16 | 52 | 25,58723765 | 52,64285714 | 3,213113065 | 0,286922299 | 0,099225766 | 1,110156088 | 2,17230401 | 9 | 99,74% |
| E15B | 7155 | 29 | 44 | 10,3285263 | 63,01239067 | 5,192066615 | 0,951333464 | 0,502595996 | 2,01043818 | 3,852616294 | 15 | 99,79% |
| E15C | 5213 | 30 | 51 | 14,73339544 | 66,04545455 | 4,678798436 | 0,868467348 | 0,451666581 | 1,823707921 | 4,212499916 | 15 | 99,71% |
| E16A | 20268 | 22 | 67 | 30,16929842 | 38,08024691 | 3,243175995 | 0,68519179 | 0,32966217 | 1,491785119 | 2,437407213 | 10 | 99,95% |
| E16B | 5439 | 27 | 29,625 | 2,824883916 | 33,30732878 | 2,829235059 | 1,21387025 | 0,586655974 | 2,419292253 | 3,702116187 | 7 | 99,87% |
| E16C | 6501 | 26 | 30,2 | 4,332882435 | 31,91368673 | 2,755393025 | 0,765137682 | 0,34189697 | 1,519518913 | 3,447028419 | 7 | 99,89% |
| E17A | 5405 | 33 | 39,875 | 5,561824197 | 46,54026548 | 3,829280012 | 1,557233532 | 0,726095223 | 3,650903833 | 4,678945051 | 11 | 99,80% |
| E17B | 12772 | 40 | 51 | 8,469443989 | 56,95763617 | 3,78960829 | 1,491428578 | 0,585360765 | 2,411735105 | 5,112679007 | 12 | 99,91% |
| E17C | 16448 | 29 | 114,5 | 59,32153707 | 112,0793269 | 2,435403501 | 1,609286439 | 0,75319082 | 4,051713154 | 3,420459927 | 19 | 99,88% |
| E18A | 4260 | 28 | 39,14285714 | 8,224251629 | 52,8309949 | 4,234452438 | 1,214767565 | 0,651412088 | 2,868716798 | 4,018982018 | 13 | 99,69% |
| E18B | 597 | 21 | 66,5 | 34,50471936 | 75,77043478 | 3,004571213 | 0,884414211 | 0,375198157 | 1,600507443 | 4,238288769 | 14 | 97,65% |
| E18C | 4055 | 24 | 29,25 | 5,360853352 | 31,90464592 | 3,005510541 | 0,969721151 | 0,53553005 | 2,152991813 | 3,385509804 | 7 | 99,83% |
| E19A | 10710 | 53 | 72,125 | 12,04541672 | 99,87885343 | 6,109370104 | 1,960380891 | 0,746046201 | 3,937724115 | 7,263510293 | 18 | 99,83% |
| E19B | 4097 | 44 | 55 | 8,470119792 | 62,89010989 | 3,990637393 | 2,3118448 | 0,847618071 | 6,562457947 | 6,885461104 | 12 | 99,71% |
| E19C | 15586 | 39 | 58,5 | 14,73186267 | 59,41216066 | 3,999319756 | 1,55635429 | 0,695135038 | 3,280140794 | 4,826549996 | 13 | 99,92% |
| E20A | 3041 | 44 | 72,875 | 16,69208316 | 86,76212287 | 5,530209465 | 1,208325009 | 0,533369177 | 2,143021742 | 7,289759735 | 22 | 99,28% |
| E20B | 1963 | 20 | 26 | 5,380028953 | 40,61416638 | 3,914357288 | 0,675902418 | 0,339904639 | 1,514932629 | 3,099549961 | 9 | 99,54% |
| E20C | 1843 | 20 | 27 | 6,635691931 | 30,24731183 | 2,704768965 | 1,145015327 | 0,593522616 | 2,460161473 | 3,135795642 | 8 | 99,57% |
| E21A | 10783 | 25 | 27 | 2,875324279 | 27,74665459 | 2,60100026 | 0,815804017 | 0,453916985 | 1,831223409 | 3,060998899 | 4 | 99,96% |
| E21B | 1655 | 13 | 16,33333333 | 4,101339739 | 20,12031558 | 2,263596699 | 0,800485185 | 0,482712644 | 1,933161498 | 1,923480549 | 5 | 99,70% |
| E21C | 9856 | 21 | 23,5 | 3,151747236 | 25,3361879 | 2,501951526 | 1,012950358 | 0,53595542 | 2,154965372 | 2,541324317 | 5 | 99,95% |
| E22A | 11902 | 31 | 40 | 8,033888185 | 42,25652855 | 3,307832523 | 0,655989617 | 0,264452461 | 1,359531433 | 3,858337101 | 9 | 99,92% |
| E22B | 1975 | 36 | 114 | 45,23954275 | 53,44207903 | 3,930021547 | 2,124743338 | 0,833110591 | 5,991991987 | 6,251443266 | 13 | 99,34% |
| E22C | 10130 | 19 | 31 | 10,73658202 | 35,66507177 | 2,451786069 | 0,823054316 | 0,442421979 | 1,793470981 | 2,259684207 | 9 | 99,91% |
| E23A | 12740 | 49 | 55 | 5,385797822 | 55,26093111 | 3,660882631 | 2,092330601 | 0,817734992 | 5,486516646 | 6,457803705 | 9 | 99,93% |
| E23B | 23719 | 35 | 54,5 | 14,7300635 | 58,28095484 | 4,380598296 | 0,976861375 | 0,429984413 | 1,754337991 | 4,032323681 | 13 | 99,95% |
| E23C | 4170 | 39 | 54 | 9,97591138 | 63,54436141 | 4,337040898 | 1,925041104 | 0,81743112 | 5,47738475 | 5,950981887 | 16 | 99,62% |
| E24A | 12973 | 19 | 19 | 0,081110711 | 19,38461538 | 2,148803731 | 1,10763424 | 0,503505169 | 2,01411966 | 2,186826885 | 1 | 99,99% |
| E24B | 6797 | 25 | 25,5 | 1,290519731 | 25,61666917 | 2,476954173 | 1,862651856 | 0,798179164 | 4,954889782 | 3,27262609 | 2 | 99,97% |
| E24C | 2025 | 18 | 40,5 | 19,23588548 | 59,38473167 | 4,114603309 | 0,956024744 | 0,450659595 | 1,820364914 | 2,721749632 | 10 | 99,51% |
| E25A | 24614 | 35 | 39,66666667 | 4,487622891 | 43,05344342 | 3,392813494 | 1,066090278 | 0,489625487 | 1,959345491 | 4,012979287 | 8 | 99,97% |
| E25B | 14174 | 35 | 56 | 14,73532563 | 63,12140422 | 4,434499857 | 1,103204967 | 0,532319893 | 2,138213673 | 4,323432407 | 15 | 99,89% |
| E25C | 7211 | 44 | 53,33333333 | 8,849914556 | 49,13864794 | 3,387721469 | 2,156671802 | 0,847112132 | 6,540741361 | 6,237974632 | 8 | 99,89% |
| E26A | 12133 | 29 | 47 | 16,08942761 | 41,63129333 | 3,380734756 | 1,420063793 | 0,591283839 | 2,446685731 | 3,565913857 | 9 | 99,93% |
| E26B | 11339 | 27 | 38,25 | 9,537045878 | 53,39053254 | 4,116322935 | 1,081652332 | 0,504307933 | 2,017381488 | 3,318231763 | 10 | 99,91% |
| E26C | 463 | 14 | 17 | 4,139124659 | 18,84099174 | 2,083236743 | 1,27613398 | 0,543670027 | 2,191396706 | 2,722692782 | 4 | 99,14% |
| E27A | 2298 | 25 | 28,75 | 4,198978428 | 30,09943552 | 2,718426833 | 1,384919541 | 0,631129654 | 2,710979644 | 3,921563406 | 6 | 99,74% |
| E27B | 5090 | 25 | 30,25 | 5,361357735 | 31,62364824 | 2,769583052 | 1,180645404 | 0,602518286 | 2,515839004 | 3,422102793 | 7 | 99,86% |
| E27C | 4665 | 36 | 78 | 25,99685632 | 77,2877479 | 4,360122359 | 0,395170971 | 0,135272106 | 1,156433147 | 5,310250563 | 21 | 99,55% |
| E28A | 14815 | 45 | 71,25 | 18,74991776 | 77,24757456 | 4,671442193 | 1,775570139 | 0,698356358 | 3,315170157 | 5,726104277 | 15 | 99,90% |
| E28B | 6032 | 38 | 54,5 | 12,89006645 | 59,32972778 | 4,377338516 | 1,628166275 | 0,698869788 | 3,320822552 | 5,415887358 | 12 | 99,80% |
| E28C | 36316 | 59 | 79 | 13,49287461 | 85,04484178 | 4,779694222 | 2,015116337 | 0,816921284 | 5,462131391 | 6,883632074 | 16 | 99,96% |
| E29A | 21938 | 28 | 96 | 48,63971901 | 72,31335417 | 4,059190206 | 0,421619198 | 0,23395409 | 1,305404789 | 3,166127669 | 17 | 99,92% |
| E29B | 7032 | 22 | 31,33333333 | 8,835210262 | 40,25777778 | 3,761240332 | 1,183984711 | 0,540508464 | 2,176318651 | 2,811539756 | 8 | 99,89% |
| E29C | 6786 | 29 | 80 | 35,16390195 | 84,55131579 | 5,276067435 | 0,248218277 | 0,083765843 | 1,091424057 | 3,884123745 | 18 | 99,73% |
| E30A | 15974 | 34 | 45 | 8,867243089 | 49,84350527 | 3,762554429 | 1,076308517 | 0,575108725 | 2,35354327 | 4,113934073 | 11 | 99,93% |

| | | | | | | | | | | | | |
|---|---|---|---|---|---|---|---|---|---|---|---|---|
| E30B | 37910 | 43 | 128,5 | 59,33962954 | 106,0655271 | 5,886966619 | 1,685829933 | 0,687738936 | 3,202448577 | 4,790281924 | 19 | 99,95% |
| E30C | 3396 | 46 | 82,14285714 | 20,81255208 | 95,81189495 | 6,21176222 | 1,340131054 | 0,603208444 | 2,520214924 | 7,522973078 | 23 | 99,32% |
| E31A | 1342 | 30 | 49,5 | 14,72740522 | 65,11989796 | 5,64014213 | 1,806834997 | 0,723158264 | 3,61217212 | 5,443041392 | 13 | 99,03% |
| E31B | 4289 | 36 | 51,6 | 11,63653675 | 60,62777778 | 3,785335916 | 1,390188782 | 0,614752363 | 2,595732987 | 5,388592745 | 13 | 99,70% |
| E31C | 24322 | 51 | 67,5 | 12,89440286 | 72,18816568 | 4,155831489 | 2,195893408 | 0,828173662 | 5,819829566 | 6,15823026 | 12 | 99,95% |
| E33A | 10051 | 44 | 53,16666667 | 7,376469556 | 54,77015082 | 3,395732399 | 1,603458616 | 0,700949444 | 3,343916204 | 5,915175746 | 11 | 99,89% |
| E33B | 37663 | 33 | 46,2 | 10,20976156 | 51,21052632 | 3,808679155 | 1,378162394 | 0,693997814 | 3,26795051 | 3,561246954 | 12 | 99,97% |
| E33C | 442 | 22 | 31,33333333 | 8,835210262 | 34,25757448 | 3,227993474 | 1,717467621 | 0,698204377 | 3,313500678 | 4,867630492 | 8 | 98,19% |
| E35A | 14439 | 21 | 26 | 6,001802614 | 29,35261708 | 2,122066203 | 1,248934497 | 0,552450508 | 2,234389755 | 2,41484188 | 5 | 99,97% |
| E35B | 12488 | 13 | 27 | 13,13392554 | 49 | 0,977066577 | 1,304969542 | 0,712599034 | 3,479459428 | 1,432827011 | 8 | 99,94% |
| E35C | 15818 | 29 | 59 | 20,91579162 | 98,03194103 | 7,839008322 | 1,074029373 | 0,604864577 | 2,530777911 | 3,438418784 | 16 | 99,90% |
| E36A | 10656 | 33 | 60,2 | 18,16198125 | 66,43759398 | 3,350244083 | 1,456652503 | 0,687405011 | 3,199027611 | 4,211027886 | 17 | 99,84% |
| E36B | 8282 | 15 | 36 | 17,32271309 | 30,03409091 | 2,921455937 | 0,984117393 | 0,568742954 | 2,318802695 | 1,775604155 | 7 | 99,92% |
| E36C | 13929 | 17 | 26 | 8,024339477 | 31,78393352 | 3,010521728 | 0,68530814 | 0,476355301 | 1,909691824 | 1,911417701 | 9 | 99,94% |
| R01A | 9278 | 117 | 135,4 | 9,916767976 | 133,2745806 | 5,499270679 | 3,083266495 | 0,925272327 | 13,38192354 | 18,87166351 | 24 | 99,74% |
| R01B | 21635 | 171 | 185,8823529 | 8,211047585 | 187,8402189 | 6,747197877 | 3,420208496 | 0,933058933 | 14,93851306 | 25,32841473 | 23 | 99,89% |
| R01C | 9929 | 102 | 114,3636364 | 7,950798514 | 114,6573209 | 5,326916589 | 2,809707674 | 0,889652336 | 9,062267038 | 15,83163102 | 17 | 99,83% |
| R02A | 6358 | 125 | 150 | 13,2999256 | 143,0509927 | 5,864998466 | 3,322988317 | 0,937898382 | 16,10263997 | 22,05621197 | 25 | 99,61% |
| R02B | 2636 | 103 | 116,125 | 7,594310164 | 115,3327365 | 5,002533422 | 3,069603884 | 0,914992252 | 11,76363353 | 21,3518024 | 21 | 99,20% |
| R02C | 3233 | 82 | 128 | 26,59559213 | 104,2625158 | 5,110898765 | 2,706521739 | 0,869709975 | 7,675184623 | 15,30506326 | 24 | 99,26% |
| R03A | 8354 | 84 | 99 | 8,655445914 | 99,88810597 | 4,748055238 | 1,645644049 | 0,634949367 | 2,739346022 | 12,9867786 | 21 | 99,75% |
| R03B | 15084 | 115 | 140,6666667 | 14,70246664 | 135,0858803 | 5,799322533 | 2,850585266 | 0,901105256 | 10,11176082 | 16,92781338 | 22 | 99,85% |
| R03C | 10645 | 111 | 136 | 13,01362107 | 135,2666215 | 5,915297315 | 2,480383488 | 0,823361762 | 5,661288354 | 17,27584023 | 26 | 99,76% |
| R04A | 17385 | 78 | 123,1111111 | 23,16353155 | 121,0383639 | 6,453693336 | 1,497198424 | 0,624639097 | 2,664102714 | 10,5261687 | 29 | 99,83% |
| R04B | 3586 | 93 | 105,6666667 | 7,511875035 | 107,8718899 | 5,027210779 | 3,048174773 | 0,919358421 | 12,40055082 | 17,44680475 | 20 | 99,44% |
| R04C | 10298 | 116 | 126 | 6,003652027 | 130,4784085 | 5,567585582 | 2,886447562 | 0,907684805 | 10,8324529 | 18,31446843 | 20 | 99,81% |
| R05A | 13630 | 69 | 75 | 4,698408382 | 78,03583169 | 4,305580592 | 2,115503727 | 0,817685224 | 5,485018929 | 9,49049318 | 12 | 99,91% |
| R05B | 6290 | 65 | 90 | 13,2982938 | 93,13652249 | 5,094876634 | 1,281319621 | 0,5183391 | 2,076149425 | 10,099738 | 25 | 99,60% |
| R05C | 6003 | 89 | 114,6666667 | 14,70155674 | 106,362275 | 5,080154101 | 1,904814642 | 0,648115667 | 2,841842917 | 14,81673362 | 22 | 99,63% |
| R06A | 4250 | 119 | 138,1176471 | 9,890714649 | 139,8569132 | 5,82606171 | 3,180428463 | 0,924607668 | 13,26394835 | 22,72490475 | 26 | 99,39% |
| R06B | 11091 | 112 | 130,0714286 | 9,955134534 | 130,1129292 | 5,607229799 | 2,304890129 | 0,754012748 | 4,065251315 | 17,32927116 | 23 | 99,79% |
| R06C | 9117 | 106 | 121,1111111 | 9,733747041 | 116,8497778 | 5,326422248 | 2,645724918 | 0,878728288 | 8,245946083 | 16,83539976 | 17 | 99,81% |
| R07A | 11226 | 145 | 166,75 | 10,46274201 | 170,1529263 | 6,35120335 | 3,150080716 | 0,925972494 | 13,50849239 | 23,49606715 | 30 | 99,73% |
| R07B | 9524 | 129 | 152,0769231 | 12,23257356 | 156,1828593 | 6,41279646 | 3,531301587 | 0,952483158 | 21,04516982 | 21,09684187 | 25 | 99,74% |
| R07C | 8072 | 105 | 126,2307692 | 11,47216818 | 132,5737668 | 5,954499423 | 3,134237124 | 0,926745361 | 13,65101258 | 17,03759298 | 24 | 99,70% |
| R08A | 6675 | 106 | 165,5 | 28,18424653 | 152,9705683 | 6,53223623 | 2,643187565 | 0,853025583 | 6,803905208 | 17,89195739 | 35 | 99,48% |
| R08B | 5300 | 52 | 86,2 | 21,92182093 | 81,79136099 | 5,000025398 | 1,70972993 | 0,724530438 | 3,630165134 | 8,003596375 | 19 | 99,64% |
| R08C | 6665 | 82 | 101 | 11,28967372 | 99,39835328 | 4,98817763 | 2,079254185 | 0,740608738 | 3,85517998 | 13,1639944 | 20 | 99,70% |
| R09A | 19546 | 99 | 122,625 | 11,78975404 | 128,1712183 | 5,999841714 | 1,728463796 | 0,680643878 | 3,131300549 | 13,61791383 | 28 | 99,86% |
| R09B | 5439 | 71 | 114,5 | 21,7848253 | 115,4239268 | 5,703873509 | 1,632909508 | 0,627819868 | 2,686870992 | 11,5285122 | 30 | 99,45% |
| R09C | 23116 | 83 | 108,6666667 | 14,70128492 | 106,2356989 | 5,004809076 | 1,679095862 | 0,601177451 | 2,507380796 | 10,82588745 | 22 | 99,90% |
| R10A | 2492 | 75 | 115,625 | 22,02071609 | 111,523735 | 5,535739608 | 2,753434294 | 0,879095288 | 8,270976232 | 14,56950382 | 26 | 98,96% |
| R10B | 3106 | 68 | 118,1428571 | 27,28133948 | 103,3418519 | 5,34230273 | 2,485910563 | 0,871591614 | 7,787653396 | 12,28106935 | 27 | 99,13% |
| R10C | 3013 | 81 | 98,5 | 10,10570542 | 99,47676623 | 5,001610166 | 2,612453504 | 0,869982702 | 7,691284305 | 15,32215256 | 21 | 99,30% |
| R11A | 8943 | 72 | 91,09090909 | 11,04845282 | 95,80979828 | 5,114113942 | 1,943570677 | 0,791252947 | 4,790486801 | 10,69886863 | 21 | 99,77% |
| R11B | 678 | 49 | 57,27272727 | 5,914548136 | 60,68199414 | 3,697303731 | 2,653669354 | 0,883863698 | 8,61057206 | 12,12355408 | 14 | 97,94% |
| R11C | 8161 | 80 | 99 | 11,6117998 | 97,96090319 | 5,165416292 | 2,277944738 | 0,823535315 | 5,666856237 | 12,3112641 | 19 | 99,77% |
| R12A | 5277 | 100 | 109,5 | 5,856225767 | 113,2766038 | 5,1223882 | 2,038411404 | 0,680840827 | 3,133232833 | 17,50765748 | 19 | 99,64% |
| R12B | 12261 | 78 | 93,54545455 | 9,444774675 | 93,61247456 | 4,96249612 | 1,986959086 | 0,781296447 | 4,572399424 | 11,13490104 | 19 | 99,85% |
| R12C | 3826 | 77 | 86,71428571 | 6,29025794 | 90,39260157 | 4,681145507 | 2,563960851 | 0,873105549 | 7,880565228 | 13,6547909 | 17 | 99,56% |
| R13A | 2505 | 109 | 122,125 | 7,594370894 | 124,062208 | 5,415529031 | 3,415996913 | 0,934838029 | 15,34637423 | 23,24493628 | 21 | 99,16% |
| R13B | 1360 | 86 | 116 | 16,15260935 | 105,0692326 | 4,77920359 | 3,214043995 | 0,927679498 | 13,8273377 | 20,40690565 | 25 | 98,16% |
| R13C | 2172 | 52 | 55,5 | 3,443146468 | 57,7378366 | 3,7842415 | 2,740642267 | 0,898887651 | 9,889988805 | 9,579674447 | 8 | 99,63% |
| R14A | 6579 | 80 | 88,66666667 | 6,413281542 | 90,12500307 | 4,690519298 | 2,485147201 | 0,771211287 | 4,370844991 | 12,81430942 | 13 | 99,80% |
| R14B | 9920 | 108 | 127,0909091 | 11,04947116 | 124,7653654 | 5,544498338 | 2,96122669 | 0,906767771 | 10,72590469 | 16,94348631 | 21 | 99,79% |
| R14C | 12306 | 124 | 133,5454545 | 6,56671508 | 132,3802 | 5,660624509 | 3,49773032 | 0,955801317 | 22,62510876 | 19,17837394 | 15 | 99,88% |
| R15A | 891 | 63 | 105 | 21,84658442 | 116,3637622 | 6,438528517 | 2,805770628 | 0,897928027 | 9,797008626 | 15,47873791 | 28 | 96,86% |
| R15B | 2914 | 69 | 100,625 | 17,96274453 | 93,10204733 | 4,893086549 | 2,442317191 | 0,855905437 | 6,939887084 | 12,68012394 | 23 | 99,21% |
| R15C | 2291 | 75 | 102,1428571 | 16,50102141 | 94,52665598 | 4,943735349 | 2,606865764 | 0,838967352 | 6,20992086 | 14,86943324 | 20 | 99,13% |
| R16A | 3438 | 80 | 108,1111111 | 15,80284133 | 105,0508944 | 5,171697285 | 2,988332591 | 0,92336244 | 13,04843218 | 14,64440716 | 23 | 99,33% |
| R16B | 2883 | 69 | 76,58333333 | 5,447539816 | 80,05742729 | 4,391244634 | 2,662707818 | 0,880061561 | 8,337610631 | 12,71057568 | 14 | 99,51% |
| R16C | 6636 | 103 | 132,25 | 15,03932773 | 130,3657895 | 5,866945951 | 2,103337746 | 0,658218492 | 2,925845832 | 17,30557251 | 27 | 99,59% |
| R17A | 34410 | 76 | 86,11111111 | 7,187932617 | 88,54329496 | 4,671829012 | 1,797269613 | 0,711914685 | 3,47119394 | 9,2429159 | 14 | 99,96% |
| R17B | 2975 | 82 | 103 | 12,20025266 | 100,5367122 | 5,009346623 | 2,571770743 | 0,802441184 | 5,061783715 | 15,60165732 | 21 | 99,29% |
| R17C | 2072 | 85 | 104,4615385 | 10,73306868 | 104,9228149 | 4,969663541 | 2,646222821 | 0,840659054 | 6,275850778 | 17,84555804 | 23 | 98,89% |
| R18A | 3028 | 67 | 86 | 11,28903049 | 92,13326252 | 5,03869194 | 2,514227636 | 0,863384501 | 7,319813699 | 12,12872145 | 20 | 99,34% |
| R18B | 4034 | 59 | 70,14285714 | 8,228611022 | 71,96703533 | 4,416870942 | 2,48852448 | 0,857858918 | 7,035263743 | 9,795809928 | 13 | 99,68% |
| R18C | 9305 | 70 | 77,2 | 6,43688376 | 75,53128205 | 4,274246012 | 2,683140677 | 0,900302848 | 10,0303768 | 10,28022649 | 9 | 99,90% |
| R19A | 15156 | 104 | 122,75 | 9,896284774 | 128,6003435 | 5,724325795 | 1,816134389 | 0,666527862 | 2,998751279 | 15,03609519 | 25 | 99,84% |
| R19B | 8465 | 73 | 98,66666667 | 14,7007584 | 97,60307151 | 5,03014559 | 2,16856077 | 0,805706915 | 5,14686357 | 10,97909872 | 22 | 99,74% |
| R19C | 55072 | 82 | 99 | 10,99195858 | 98,52177368 | 4,701279504 | 2,523515125 | 0,874123745 | 7,944310066 | 9,458248821 | 17 | 99,97% |
| R20A | 4474 | 98 | 117,25 | 10,86849522 | 115,187122 | 5,252128808 | 2,950620503 | 0,9081313 | 10,88510016 | 17,70117663 | 22 | 99,51% |
| R20B | 14133 | 108 | 127 | 11,29042867 | 124,0933659 | 5,62408837 | 2,357851135 | 0,828250331 | 5,822427519 | 15,90378854 | 20 | 99,86% |
| R20C | 21839 | 97 | 104,3333333 | 5,675938256 | 103,7825966 | 4,99057245 | 2,139192356 | 0,811580591 | 5,30730886 | 13,0696846 | 12 | 99,95% |
| R21A | 4347 | 98 | 126,875 | 16,6964289 | 112,3648053 | 5,038659888 | 2,865532515 | 0,899468889 | 9,94716944 | 17,81378443 | 22 | 99,49% |
| R21B | 8217 | 65 | 70,5 | 4,509194704 | 73,62319339 | 4,28736378 | 1,834220777 | 0,697976864 | 3,311004624 | 9,629150294 | 11 | 99,87% |
| R21C | 4119 | 88 | 109 | 12,20046999 | 110,3803419 | 5,457818005 | 2,779120136 | 0,864064534 | 7,356431917 | 15,80842292 | 21 | 99,49% |
| R22A | 8077 | 93 | 116 | 12,46507333 | 119,0574778 | 5,686086696 | 2,098589393 | 0,725770433 | 3,646579806 | 14,74368824 | 24 | 99,70% |
| R22B | 7180 | 104 | 126,8947368 | 11,01449875 | 138,2588165 | 6,160766818 | 1,863580829 | 0,636585377 | 2,751677938 | 17,23405251 | 30 | 99,58% |
| R22C | 4224 | 84 | 93,1 | 6,483830496 | 92,61369121 | 4,625962375 | 2,442560861 | 0,82117865 | 5,592173416 | 14,85803189 | 14 | 99,67% |
| R23A | 13276 | 69 | 76,58333333 | 5,447539816 | 79,01676487 | 4,27560083 | 1,013072218 | 0,375629189 | 1,601612348 | 9,530470694 | 14 | 99,89% |
| R23B | 8348 | 93 | 111,0714286 | 9,954839829 | 110,6894711 | 5,05628615 | 1,427156515 | 0,513247896 | 2,054433852 | 14,65282949 | 23 | 99,72% |
| R23C | 1593 | 79 | 93,61538462 | 8,645497237 | 98,11727992 | 5,025164274 | 2,774908952 | 0,852427582 | 6,776334025 | 17,46131367 | 20 | 98,74% |
| R24A | 4621 | 90 | 96,5 | 4,880693758 | 96,78955658 | 4,705738285 | 2,95608743 | 0,902634169 | 10,27054348 | 15,84869007 | 13 | 99,72% |
| R24B | 3708 | 75 | 84,54545455 | 6,565921562 | 85,76513661 | 4,479032317 | 2,333622241 | 0,808592955 | 5,224468083 | 13,31443933 | 15 | 99,60% |
| R24C | 16445 | 94 | 124,6 | 20,01035177 | 113,2727213 | 5,234945728 | 2,581638754 | 0,864083636 | 7,357465811 | 13,18460336 | 18 | 99,89% |
| R25A | 8555 | 91 | 109,0714286 | 9,954802835 | 113,1117598 | 5,38535311 | 1,965570022 | 0,745095581 | 3,923039088 | 14,2151452 | 23 | 99,73% |
| R25B | 4418 | 113 | 116,4375 | 2,977329753 | 117,4819703 | 5,199789532 | 3,439758843 | 0,939983987 | 16,6622197 | 21,13158846 | 11 | 99,75% |

| | | | | | | | | | | | | |
|---|---|---|---|---|---|---|---|---|---|---|---|---|
| R25C | 6308 | 93 | 108,1111111 | 9,733396019 | 107,1719278 | 5,204324097 | 2,750841605 | 0,868828181 | 7,623588737 | 15,46542058 | 17 | 99,73% |
| R26A | 3037 | 75 | 87,75 | 7,963151454 | 91,53730459 | 4,836852906 | 2,748277461 | 0,878133576 | 8,205705598 | 13,91385568 | 18 | 99,41% |
| R26B | 762 | 37 | 43 | 5,384355028 | 43,536825 | 3,069923235 | 1,710983871 | 0,595628302 | 2,472972282 | 8,130112945 | 9 | 98,82% |
| R26C | 665 | 59 | 87,875 | 16,69382619 | 88,42367788 | 5,109097681 | 2,999870808 | 0,907519928 | 10,81314033 | 15,63539872 | 22 | 96,69% |
| R27A | 13240 | 97 | 112 | 9,979883505 | 107,6642656 | 5,106927787 | 2,460075346 | 0,831138521 | 5,922013747 | 14,18093519 | 16 | 99,88% |
| R27B | 1942 | 54 | 77,33333333 | 13,63365637 | 87,46582031 | 5,866373557 | 1,942566605 | 0,739835773 | 3,843725985 | 10,29539651 | 21 | 98,92% |
| R27C | 6530 | 82 | 103,1111111 | 12,6056254 | 105,513658 | 5,140433 | 2,38459336 | 0,830425906 | 5,897127175 | 13,21560726 | 20 | 99,69% |
| R28A | 4314 | 84 | 99 | 10,3348394 | 94,29552265 | 4,768115789 | 2,823129134 | 0,873046731 | 7,876914143 | 14,79133248 | 15 | 99,65% |
| R28B | 12048 | 73 | 82,1 | 6,483542822 | 83,22824091 | 4,466079323 | 1,074487708 | 0,368303953 | 1,583039826 | 10,33739022 | 14 | 99,88% |
| R28C | 17924 | 96 | 110,25 | 8,653564329 | 115,0656566 | 5,397769943 | 2,7725825 | 0,898454071 | 9,847760572 | 13,3239395 | 19 | 99,89% |
| R29A | 2083 | 71 | 80,1 | 6,483482028 | 80,5494898 | 4,307225672 | 2,914626926 | 0,895719619 | 9,589531473 | 14,21733509 | 14 | 99,33% |
| R29B | 14276 | 72 | 87 | 12,83615683 | 79,56927564 | 4,303077689 | 2,586243978 | 0,866944365 | 7,515653147 | 9,897214181 | 10 | 99,93% |
| R29C | 1980 | 78 | 88 | 6,656407826 | 90,94234013 | 4,725133948 | 3,171030313 | 0,932121212 | 14,73214286 | 16,20350255 | 16 | 99,19% |
| R30A | 5569 | 95 | 113,4 | 9,916471245 | 121,6743697 | 5,663503347 | 2,409998099 | 0,744745599 | 3,917660163 | 16,27135379 | 24 | 99,57% |
| R30B | 15077 | 94 | 106,6666667 | 7,51188852 | 109,7295433 | 5,173603477 | 2,140076429 | 0,788913029 | 4,737383807 | 13,37414071 | 20 | 99,87% |
| R30C | 7697 | 73 | 80 | 7,113148037 | 77,07312734 | 4,089451725 | 3,062330823 | 0,926209319 | 13,55184678 | 11,16709322 | 7 | 99,91% |
| R31A | 20193 | 90 | 109 | 11,61219993 | 109,9998234 | 5,366771468 | 1,182088877 | 0,383210938 | 1,621299829 | 12,13304458 | 19 | 99,91% |
| R31B | 14163 | 110 | 127 | 10,99298777 | 119,7194232 | 5,301003827 | 2,344949816 | 0,760737271 | 4,179505948 | 16,2437764 | 17 | 99,88% |
| R31C | 17857 | 144 | 162,4 | 9,917022259 | 163,3907578 | 6,303175189 | 3,105822566 | 0,921884324 | 12,80152778 | 21,40378945 | 24 | 99,87% |
| R32A | 6323 | 92 | 109 | 10,99238686 | 104,2475613 | 5,028951517 | 2,229830509 | 0,727566749 | 3,670623894 | 15,25911918 | 17 | 99,73% |
| R32B | 3044 | 103 | 117,0555556 | 7,767983442 | 123,1605398 | 5,564712779 | 3,104350472 | 0,911871396 | 11,34705374 | 20,58776772 | 23 | 99,24% |
| R32C | 4404 | 91 | 114,1 | 13,1422216 | 110,3722259 | 5,253172112 | 2,777600651 | 0,884984833 | 8,69450551 | 16,22935165 | 22 | 99,50% |
| R33A | 7005 | 124 | 143,1176471 | 9,89076029 | 143,2865647 | 5,814209557 | 2,905609505 | 0,88570071 | 8,748960761 | 21,40157907 | 26 | 99,63% |
| R33B | 7762 | 135 | 160,2 | 12,60087616 | 159,9077196 | 6,244001795 | 3,324771019 | 0,937103414 | 15,89911422 | 23,21510894 | 28 | 99,64% |
| R33C | 6638 | 117 | 132,8125 | 8,713464036 | 136,950796 | 5,964197069 | 2,898046801 | 0,894029609 | 9,436598194 | 20,17542161 | 23 | 99,65% |
| R34A | 18385 | 88 | 94,6 | 5,135525071 | 95,35522646 | 4,798534758 | 1,866030103 | 0,636049114 | 2,747623479 | 11,99677537 | 12 | 99,93% |
| R34B | 2334 | 89 | 101,2142857 | 7,433907955 | 104,1242564 | 5,071767361 | 3,162098517 | 0,922234485 | 12,85917022 | 18,33382712 | 19 | 99,19% |
| R34C | 3566 | 79 | 89,90909091 | 7,244322832 | 90,36197921 | 4,566980015 | 2,606642768 | 0,850384969 | 6,683820414 | 14,30484601 | 16 | 99,55% |
| R35A | 5722 | 91 | 103,75 | 7,96351396 | 105,6801425 | 5,170300101 | 2,759191604 | 0,896308648 | 9,644005811 | 15,36465283 | 18 | 99,69% |
| R35B | 4577 | 106 | 125,7142857 | 10,63362235 | 127,1532815 | 5,728867387 | 2,958007737 | 0,906436888 | 10,68797279 | 19,38335349 | 24 | 99,48% |
| R35C | 6347 | 98 | 123 | 13,29938832 | 120,335061 | 5,498278297 | 2,554748164 | 0,850995927 | 6,711225962 | 16,44816562 | 25 | 99,61% |
| R36A | 3747 | 98 | 140 | 21,84869406 | 123,0118385 | 5,540477239 | 2,769163189 | 0,882310403 | 8,496927733 | 18,42054054 | 28 | 99,25% |
| R36B | 4929 | 78 | 102,4285714 | 15,16510634 | 95,96821766 | 4,867221256 | 2,711474358 | 0,883688651 | 8,597613268 | 13,15646819 | 19 | 99,61% |
| R36C | 3816 | 83 | 86,92857143 | 3,336842192 | 88,61115869 | 4,470307979 | 2,704308635 | 0,876254236 | 8,081084681 | 14,96867649 | 11 | 99,71% |

Table S3. Physicochemical properties of the soil from the World Olive Germplasm Collection (Córdoba, Spain)

| **Parameter** | **Mean values[1]** |
|---|---|
| Cation Exchange Capacity (meq /100 g) | 13.97 ± 1.35 |
| Calcium (mEq /100 g) | 9.42 ± 1.30 |
| Magnesium (mEq /100 g) | 3.33 ± 0.36 |
| Sodium (mEq /100 g) | 0.32 ± 0.02 |
| Potassium (mEq /100 g) | 0.90 ± 0.08 |
| Carbonates (%) | 15.93 ± 2.11 |
| Active lime (%) | 2.06 ± 0.35 |
| Assimilable phosphorus (p.p.m.) | 26.27 ± 3.28 |
| Organic matter (%) | 1.05 ± 0.17 |
| Organic nitrogen (%) | 0.09 ± 0.01 |
| pH 1 / 2.5 | 8.43 ± 0.06 |
| pH (in KCl) | 7.54 ± 0.04 |
| Exchangeable potassium (p.p.m.) | 380.5 ± 32.2 |
| Clay (%) | 22.16 ± 1.77 |
| Sand (%) | 40.97 ± 2.49 |
| Silt (%) | 36.87 ± 1.12 |

[1]Based on 10 samples (± standard error)
